# Supplementary material for: Health professions faculty’s perceptions of online teaching and learning during the COVID-19 pandemic
Source: PLoS One. 2022 Nov 17;17(11):e0276170. doi: 10.1371/journal.pone.0276170 (PMC9671340; doi:10.1371/journal.pone.0276170)
Supplement: Attachment — Submitted filename: PONE-D-21-33695_R1 with comments.pdf [file pone.0276170.s004.pdf]

PLOS ONE

Health professions faculty’s perceptions of online teaching and learning during the COVID-19 pandemic

--Manuscript Draft--

**Manuscript Number:**	PONE-D-21-33695R1
**Article Type:**	Research Article
**Full Title:**	Health professions faculty’s perceptions of online teaching and learning during the COVID-19 pandemic
**Short Title:**	Health professions educators' perceptions online teaching under COVID-19 pandemic
**Corresponding Author:**	Midion Mapfumo Chidzonga, BDS FFDRCSI<br>University of Zimbabwe Faculty of Medicine: University of Zimbabwe College of Health Sciences<br>Harare, n/a ZIMBABWE
**Keywords:**	COVID-19 pandemic; higher education; online teaching, learning and assessment, health professions faculty
**Abstract:**	The global societal impact of the COVID-19 pandemic is incalculable with profound social suffering, deep economic hardships and enforced closure of schools, businesses, and higher learning institutions through the imposition of lockdown and social distancing in mitigation of the spread of the SARS-Cov-2 infection. Institutions have had to hastily migrate teaching, learning and assessment to online domains, at times with ill-prepared academics, students and institutions and with unwelcome and disorienting consequences. Our study surveyed perspectives of faculty at the University of Zimbabwe Faculty of Medicine and Health Sciences (UZFMHS) towards the hastily adopted online teaching, learning and assessment implemented in response to the mitigation of the COVID-19 pandemic. Twenty nine (29) faculty in all the major disciplines and career hierarchy. There were mixed responses regarding the use of this modality for teaching, learning and assessment: training before online teaching, learning and assessment, advantages and disadvantages, cost effectiveness, effectiveness for teaching, learning and assessment, effect on student feedback, disruptions from internet connectivity issues, interaction with students, suitability for practical training, and barriers to online teaching, learning and assessment. These results would enable the UZFMHS develop institutional and personalised approaches that would enable execution of online teaching, learning and assessment under the current and post COVID-19 pandemic.
**Order of Authors:**	Midion Mapfumo Chidzonga, BDS FFDRCSI
	Clara Opha Haruzivishe, PhD
	Judith Rukweza, PhD
	Vasco Chikasha, PhD
**Response to Reviewers:**	Faculty research Response to First reviewer comments<br><br>1.How many were invited? Give the number please.<br><br>We used a convenient sample of 100 faculty from the above disciplines. The faculty approached where those who were accessible on campus during the time of data collection. Only 29 faculty responded.<br><br>2.was the questionnaire validated? Totally how many questions were there related to the demographic information and how many related to online teaching and learning?<br><br>The tool used to collect data was piloted on 5 faculty who were excluded from the main study. Following this pilot some items were removed and only relevant items were left: three 3 questions relating to demographic data and 13 questions relating to online teaching and learning were finally used for the study.<br><br>3.This has already been described in the introduction.

Powered by Editorial Manager® and ProduXion Manager® from Aries Systems Corporation

	This has already been described in the introduction.<br><br>4.what is the relevance of this to the study carried out?<br><br>This will be removed.<br><br>5.What percentage of the total faculty members participated?<br><br>we got responses from 29 out of 100 members approached.<br><br>6.It is saying the same thing as the previous paragraph<br><br>This will be removed<br><br>7.As per the table 1, 48% agreed while 52% disagreed, that makes this presumption of "majority" of the faculty, invalid<br><br>This has been rephrased accordingly: The majority of faculty did not agreed that online teaching enhanced their teaching capacity while those who believed in the enhancement from online teaching felt that it added value to the learning of the students<br><br>Reviewers comments from second reviewer<br><br>1.How many were invited? Give the number please.<br>a.100 faculty were invited<br><br>2.was the questionnaire validated? Totally how many questions were there related to the demographic information and how many related to online teaching and learning?<br><br>a.Yes. The questionnaires were developed using relevant items that were used in literature. The tool was then piloted on 5 faculty who were excluded from the main study. Following this pilot some items were removed and only relevant items were left: three 3 questions relating to demographic data and 13 questions relating to online teaching and learning were finally used for the study.<br><br>3.This has already been described in the introduction.<br>a.Agreed. this has been removed.<br><br>4.what is the relevance of this to the study carried out?<br>a.Nor relevant. This has been removed.<br><br>5.What percentage of the total faculty members participated?<br><br>a.29 out of 100<br>6.It is saying the same thing as the previous paragraph<br>a.This will be removed<br><br>7.As per the table 1, 48% agreed while 52% disagreed, that makes this presumption of "majority" of the faculty, invalid<br><br>a.In agreement. This has been rephrased as follows: "The majority of faculty did not agree that on-line teaching enhanced their teaching capacity while those who believed in the enhancement from on-line teaching felt that it added value to the learning of the students. "
**Additional Information:**	
**Question**	**Response**
**Financial Disclosure**	1.All authors were under the same funding: initials are MMC, COH, JR, VC

Enter a financial disclosure statement that describes the sources of funding for the work included in this submission. Review the submission guidelines for detailed requirements. View published research articles from *PLOS ONE* for specific examples.<br><br>This statement is required for submission and **will appear in the published article** if the submission is accepted. Please make sure it is accurate.<br><br>**Unfunded studies**<br>Enter: *The author(s) received no specific funding for this work.*<br><br>**Funded studies**<br>Enter a statement with the following details:<br>• Initials of the authors who received each award<br>• Grant numbers awarded to each author<br>• The full name of each funder<br>• URL of each funder website<br>• Did the sponsors or funders play any role in the study design, data collection and analysis, decision to publish, or preparation of the manuscript?<br>• **NO** - Include this sentence at the end of your statement: *The funders had no role in study design, data collection and analysis, decision to publish, or preparation of the manuscript.*<br>• **YES** - Specify the role(s) played.<br><br>* typeset	2.www.norad.no<br>3.Funded by the Norwegian Programme for Capacity Development in Higher Education and Research Development (NORHED)<br><br>The funders had no role in study design, data collection and analysis, decision to publish, or preparation of the manuscript.
**Competing Interests**<br><br>Use the instructions below to enter a competing interest statement for this submission. On behalf of all authors, disclose any competing interests that could be perceived to bias this work—acknowledging all financial support and any other relevant financial or non-financial competing interests.<br><br>This statement is required for submission and **will appear in the published article** if	The authors have declared that no competing interests exist.

Powered by Editorial Manager® and ProduXion Manager® from Aries Systems Corporation

the submission is accepted. Please make sure it is accurate and that any funding sources listed in your Funding Information later in the submission form are also declared in your Financial Disclosure statement.<br><br>View published research articles from *PLOS ONE* for specific examples.<br><br>**NO authors have competing interests**<br><br>Enter: *The authors have declared that no competing interests exist.*<br><br>**Authors with competing interests**<br><br>Enter competing interest details beginning with this statement:<br><br>*I have read the journal's policy and the authors of this manuscript have the following competing interests: [insert competing interests here]*<br><br>* typeset	
**Ethics Statement**<br><br>Enter an ethics statement for this submission. This statement is required if the study involved:<br><br>• Human participants<br>• Human specimens or tissue<br>• Vertebrate animals or cephalopods<br>• Vertebrate embryos or tissues<br>• Field research<br><br>Write "N/A" if the submission does not require an ethics statement.<br><br>General guidance is provided below. Consult the submission guidelines for detailed instructions. **Make sure that all information entered here is included in the Methods section of the manuscript.**	Conflicts of interest/Competing interest: None<br>Availability of data and material (data transparency): Available<br>Code availability (software application or custom code): Not applicable<br>Authors' contributions (optional)<br>Ethics approval: Permission to carry out the study at the University of Zimbabwe Faculty of Medicine and Health Sciences (UZFMHS) was granted by the Dean of the UZFMHS. Ethical clearance to carry out the study was obtained from the local ethical review board, Joint Research Ethics Committee of the UZFMHS and Parirenyatwa Group of Hospital (JREC) and the Medical Research Council of Zimbabwe (MRCZ).Informed consent was obtained from the participants before participation in the study. No risks to the participants were envisaged during the study process. Benefits to the participants may accrue through their perspectives being used to enrich the implementation of online teaching, learning and assessment.<br>Participation was voluntary and participants were requested to read the consent form along with the information sheet and only proceed with filling in the form if they were agreeable with the information given on the consent form and the information sheet.<br>Consent to participate: Participation was voluntary and participants were requested to read the consent form along with the information sheet and only proceed with filling in the form if they were agreeable with the information given on the consent form and the information sheet.<br>Consent for publication: Not applicable<br>Text formatting: 10-point Times Roman<br><br>Acknowledgements: Many thanks to the NORHED for funding this project.

Powered by Editorial Manager® and ProduXion Manager® from Aries Systems Corporation

**Format for specific study types**<br><br>**Human Subject Research (involving human participants and/or tissue)**<br>• Give the name of the institutional review board or ethics committee that approved the study<br>• Include the approval number and/or a statement indicating approval of this research<br>• Indicate the form of consent obtained (written/oral) or the reason that consent was not obtained (e.g. the data were analyzed anonymously)<br><br>**Animal Research (involving vertebrate animals, embryos or tissues)**<br>• Provide the name of the Institutional Animal Care and Use Committee (IACUC) or other relevant ethics board that reviewed the study protocol, and indicate whether they approved this research or granted a formal waiver of ethical approval<br>• Include an approval number if one was obtained<br>• If the study involved *non-human primates*, add *additional details* about animal welfare and steps taken to ameliorate suffering<br>• If anesthesia, euthanasia, or any kind of animal sacrifice is part of the study, include briefly which substances and/or methods were applied<br><br>**Field Research**<br><br>Include the following details if this study involves the collection of plant, animal, or other materials from a natural setting:<br>• Field permit number<br>• Name of the institution or relevant body that granted permission	
**Data Availability**<br><br>Authors are required to make all data underlying the findings described fully available, without restriction, and from the time of publication. PLOS allows rare exceptions to address legal and ethical concerns. See the PLOS Data Policy and FAQ for detailed information.	Yes - all data are fully available without restriction

A Data Availability Statement describing where the data can be found is required at submission. Your answers to this question constitute the Data Availability Statement and **will be published in the article**, if accepted.<br><br>**Important:** Stating 'data available on request from the author' is not sufficient. If your data are only available upon request, select 'No' for the first question and explain your exceptional situation in the text box.<br><br>Do the authors confirm that all data underlying the findings described in their manuscript are fully available without restriction?	
**Describe where the data may be found in full sentences. If you are copying our sample text, replace any instances of XXX with the appropriate details.**<br><br>• If the data are **held or will be held in a public repository**, include URLs, accession numbers or DOIs. If this information will only be available after acceptance, indicate this by ticking the box below. For example: *All XXX files are available from the XXX database (accession number(s) XXX, XXX.*).<br>• If the data are all contained **within the manuscript and/or Supporting Information files**, enter the following: *All relevant data are within the manuscript and its Supporting Information files.*<br>• If neither of these applies but you are able to provide **details of access elsewhere**, with or without limitations, please do so. For example:<br><br>*Data cannot be shared publicly because of [XXX]. Data are available from the XXX Institutional Data Access / Ethics Committee (contact via XXX) for researchers who meet the criteria for access to confidential data.*<br><br>*The data underlying the results presented in the study are available from (include the name of the third party*	Not applicable

and contact information or URL).<br>• This text is appropriate if the data are owned by a third party and authors do not have permission to share the data.<br><br>* typeset	
Additional data availability information:	Tick here if your circumstances are not covered by the questions above and you need the journal's help to make your data available.

Powered by Editorial Manager® and ProduXion Manager® from Aries Systems Corporation

Click here to access/download;Manuscript;Health Professions COVID-19.docx

TITLE PAGE

Title: Health professions faculty's perceptions of online teaching and learning during the COVID-19 pandemic

1. Midion Mapfumo CHIDZONGA

Department of Oral Health

Faculty of Medicine and Health Sciences

University of Zimbabwe

HARARE

ZIMBABWE

mtmchidzonga@yahoo.com

ORCID http://orcid.org/0000-0002-4574-1158

2. Clara HARUZIVISHE

Department of Primary Health Care

Unit of Nursing Sciences

Faculty of Medicine and Health Sciences

University of Zimbabwe

HARARE

ZIMBABWE

claraopha@gmail.com

3. Vasco CHIKWASHA

Unit of Global and Public Health

Department of Primary Health Care

Faculty of Medicine and Health Sciences

University of Zimbabwe

HARARE

ZIMBABWE

vchikasha@gmail.com

4. Judith RUKWEZA

Nursing Sciences

Department Primary Health Care

Faculty of Medicine and Health Sciences

University of Zimbabwe

HARARE

ZIMBABWE

rukwezajudith@gmail.com

Corresponding Author: Midion Mapfumo Chidzonga

mtmchidzonga@yahoo.com

ABSTRACT

The global societal impact of the COVID-19 pandemic is incalculable with profound social suffering, deep economic hardships and enforced closure of schools, businesses, and higher learning institutions through the imposition of lockdown and social distancing in mitigation of the spread of the SARS-Cov-2 infection. Institutions have had to hastily migrate teaching, learning and assessment to online domains, at times with ill-prepared academics, students and institutions and with unwelcome and disorienting consequences. Our study surveyed perspectives of faculty at the University of Zimbabwe Faculty of Medicine and Health Sciences (UZFMHS)

towards the hastily adopted online teaching, learning and assessment implemented in response to the mitigation of the COVID-19 pandemic. Twenty nine (29) faculty in all the major disciplines and career hierarchy. There were mixed responses regarding the use of this modality for teaching, learning and assessment: training before online teaching, learning and assessment, advantages and disadvantages ,cost effectiveness, effectiveness for teaching, learning and assessment, effect on student feedback, disruptions from internet connectivity issues, interaction with students, suitability for practical training, and barriers to online teaching, learning and assessment. These results would enable the UZFMHS develop institutional and personalised approaches that would enable execution of online teaching, learning and assessment under the current and post COVID-19 pandemic.

Keywords: COVID-19 pandemic; higher education; online teaching, learning and assessment, health professions faculty.

DECLARATIONS

Funding: Norwegian Programme for Capacity Development in Higher Education and Research Development (NORHED)

Conflicts of interest/Competing interest: Not applicable

Availability of data and material (data transparency): Available

Code availability (software application or custom code): Not applicable

Authors' contributions (optional)

Ethics approval: Permission to carry out the study at the University of Zimbabwe Faculty of Medicine and Health Sciences (UZFMHS) was granted by the Dean of the UZFMHS. Ethical clearance to carry out the study was obtained from the local ethical review board, Joint Research Ethics Committee of the UZFMHS and Parirenyatwa Group of Hospital (JREC) and the Medical Research Council of Zimbabwe (MRCZ).Informed consent was obtained from the participants before participation in the study. No risks to the participants were envisaged during

the study process. Benefits to the participants may accrue through their perspectives being used to enrich the implementation of online teaching, learning and assessment.

Participation was voluntary and participants were requested to read the consent form along with the information sheet and only proceed with filling in the form if they were agreeable with the information given on the consent form and the information sheet.

Consent to participate: Participation was voluntary and participants were requested to read the consent form along with the information sheet and only proceed with filling in the form if they were agreeable with the information given on the consent form and the information sheet.

Consent for publication: Not applicable

Text formatting: 10-point Times Roman

Automatic page numbering

Acknowledgements: Many thanks to the NORHED for funding this project.

INTRODUCTION: The World Health Organisation (WHO) declared COVID-19 a global public health emergency of international concern on 30th January 2020 and subsequently declared it a pandemic on 11th of March [1, 2]. The societal impact of COVID-19 is incalculable as the pandemic continues to cause profound social suffering and deep economic hardships especially for society's most vulnerable and less fortunate [1,2,3]. Many aspects of life have been seriously disrupted by the COVID-19 pandemic. The lockdown measures, in a bid to mitigate the spread of the SARS-Cov-2 infection, have led to enforced closure of schools, colleges, and universities, but without cessation of all learning, teaching and assessment although many forms of assessment have had to be suspended [2,4]. Most institutions of higher learning had to hastily migrate to the online domains with some of them with poor online infrastructures as well as ill-prepared academics and students [5, 6, and 7]. The new experience has been unwelcome and disorienting to some educators, and students alike. The pandemic has also exposed deficiencies in the use of technology in higher education, which has long been debated, and is now overdue [2].The University of Zimbabwe Faculty of Medicine and Health Sciences (UZFMHS) now has to embrace this change hastily. This new challenge along with the attendant obstacles has to be faced by many higher education institutions [8].There is no indication when the pandemic will be contained and as such educational institutions across the globe have now opted to use the available technical resources to create online teaching, learning and assessment material for students of all academic levels [9,10].The pandemic has thus created a forced digitalisation change, which was long overdue, and an opportunity to accelerate digital transformation, in health professions education [11,12].This is a positive move post COVID-19 pandemic. Pre-clinical health professions education can be taught through webinars. Unfortunately, online teaching, learning, and assessment is most useful for theoretical content but innovative ways have had to be developed to enable clinical training and assessment [13,14,15,16].It is known that formative and high-stakes examinations could be carried out by modifying the current assessment processes such as multiple choice questions, short-answer questions can be done online at the same time viva voce, teleconferencing between candidate and examiners; use

of simulation/ objective structured clinical examination; modify patient contact stations to simulated patients; task-trainers and hybrid simulations; patient requiring components; use of personal protective equipment (PPE) at each station; examiner view the examination process remotely .Clinical training depends heavily on hands-on-patient contact to enable clinical skills acquisition, practical work in the laboratories which would require advanced technology which is currently unavailable in most low and middle income countries (LMICs). However, collaboration with high income countries (HIC) may alleviate this situation. Innovative approaches are being put in place to enable continued clinical training: vaccination of all students and faculty; provision of adequate PPE to students ,faculty and patients; good hand hygiene; mask wearing; face shields; social distancing; full-body PPE(long-sleeved disposable gown, eye protection, gloves) for aerosol producing procedures; and small numbers of students at a time. For non-aerosol producing procedures, minimum PPE is provided (disposable apron, facemask, gloves and eye protection is provided. This ensures that the safety of patients, students and staff is not compromised.

Health professions students need to be exposed to patients in an authentic environment, an apprenticeship type of learning. This is usually in hospital wards, outpatient clinics and simulated teaching laboratories. Under the current COVID-19 pandemic live broadcasts, films or virtual case presentations, and surgical procedures might be used to complement the limited access to patients.

Faculty are expected to increase their digital competence so as to come up with innovative virtual teaching, learning, and assessment tools.

The COVID-19 pandemic has curtailed clinical placements as well as elective services offered in clinical services. However, there is need to continue to adequately train health professionals to run the healthcare system. Student clinical placement has to continue while observing safe supervision of students, adequate provision of PPE, social distancing, washing and sanitising hands. Various learning management systems are available: Moodle, Massive Open Online Courses. Several tools are also available that allow for video communications, video and audio conferencing, chats, and webinars: Skype, Zoom, and Google Hangouts Meet, Google school [13].It is possible to set up real-time classroom experience that simulates patient encounters using live HD-quality mobile or fixed-cameras that ensures that all distance learners can collaborate at an equivalent baseline [14].Current virtual learning management systems offer access to educational content from anywhere, synchronously or asynchronously with interactive simulation learning [13].Unfortunately the theoretical knowledge has the challenge of not being able to be transferred to patient management. The way forward would be to introduce interactive simulation learning (computerised simulation education) which unfortunately need massive infrastructure ranging from clinical simulation management software and hardware, design and planning tools, file backup, cloud-based eLearning, and expert teams to provide support to counsellor education, case developers, and virtual patient training [13].

This study at the UZFMHS surveyed the faculty's perspectives towards online teaching, learning and assessment. The information gathered would enable development of online approaches tailor made based the perceptions of faculty.

METHODOLOGY

SETTING: The study was conducted at the UZFMHS which provides health professions education and training to various healthcare cadres.

STUDY DESIGN: Mixed method design.

ETHICAL CONSIDERATIONS: Permission to carry out the study was obtained from the Dean of the UZFMHS. Ethical clearance was obtained from the local ethical review board, Joint Research Ethics Committee of the UZFMHS and Parirenyatwa Group of Hospital (JREC) and the Medical Research Council of Zimbabwe (MRCZ).Informed consent was obtained from the participants. Participation was voluntary. There were no risks to the participants. Participation was voluntary and participants were requested to read the consent form and only proceed with filling in the form if they were agreeable with the information given on the consent form. Benefits to the participants may accrue should their perspectives be used to enrich the development of online teaching, learning and assessment.

STUDY PARTICIPANTS: The participants were faculty of medicine, dentistry, nursing, physiotherapy, occupational therapy, pharmacy, health promotion and education, radiographers and medical laboratory scientists.

SAMPLING: We invited faculty in the above disciplines to participate.

STUDY INSTRUMENTS: An online questionnaire was created in Google Forms and emailed to faculty. The questionnaire captured demographics, academic grade, number of years in teaching, computer literacy, device used when delivering teaching type of internet connections used, whether one received training on online teaching and learning, benefits of online learning to students, role of online learning in enhancing teaching, belief in online teaching, cost effectiveness, convenience for teaching, disadvantages and advantages of online education, barriers to online teaching, and online teaching practice, and platforms used in teaching.

DATA ANALYSIS: Data collected through the online survey was exported to STATA 15.1 statistical software for cleaning, analysis, calculation frequencies and percentages for categorical data. Qualitative data is presented as themes and subthemes .Frequency responses to "YES" and "NO" items are presented in the tables.

RESULTS:

Twenty nine (29) faculty responded, 62.1% (n=18) males and 37.9% (n=11) females. The teaching experience of faculty ranged from 2 years to 44 years. Sixty five point five per cent (65.5%, n=19) had received training on online teaching and 34.5% had not received any training.

On belief in online teaching, Table 1, shows responses to "Yes and "No" questions regarding online teaching, learning and assessment with 65.5% of the respondents indicating that they had received training on online teaching, learning and assessment indicative of a possible positive participation in online teaching. However, 51.7% did not enjoy teaching online with 52.0% believing that online teaching does not enhance their teaching capacity.

QUESTIONS	YES %/N	NO %/N
Have you received training on online teaching and learning?	65.5%/19	34.5%/10
Do you enjoy online teaching and learning?	48.3%/14	51.7%/15
Do you believe in benefits of online learning to students?	93%/27	7%/2
Do you believe that online learning enhances your teaching capacity	48%/14	52%/15

Table 1. Shows answers to questions regarding online teaching and learning

Platform	N/%
Zoom	18/62.1%
Moodle	5/17.2%
E-Mhare	2/6.9%
Google meet	1/3.4%
Google classes	1/3.4%
Zoom and Google	1/3.4%
WhatsApp and emails	1/3.4%
Totals	29/100%

Table 7. Platforms used for online teaching

Those who did not believe that there were benefits in online teaching supported their views by that there were no additional benefits when compared to physical contacts with students and that students were less engaged and feedback is curtailed. They also pointed out the need for proper training on online teaching, requisite tools and internet access for both students and faculty as well as negative impact on hands on courses or skills transfer.

Table 2.Shows the academic grades of faculty. Teaching experience of faculty ranged from 2 to 44 years. All the faculty members were comfortable using a computer for teaching and learning. The majority of the faculty respondents are in the lecturer and senior lecturer grade. This is a positive situation as this group is likely to be in the age group that is technology savvy and should be able to navigate the education technology with ease.

Grade	N (%)
Professor	8 (27.6%
Associate Professor	0 (0.0%)
Senior Lecturer	10 (34.5%)
Lecturer	11 (37.9%)
TOTAL	29 (100%)

Table 2. Shows academic grade of faculty.

Table 3. Shows the devices used in delivery of teaching. The majority of faculty, 82.7%, deliver their lectures using laptops which means they could deliver their teaching at any location where there is internet connectivity.

Device	N (%)
Smartphone	0 (0%)
Tablet	1 (3.5%)
Laptop	24 (82.7%)
Personal Computer	3 (10.3%)
Other (device not indicated)	1 (3.5%)
Total	29 (100.0%)

Table3. Shows devices for delivering teaching.

Table 4. Shows that most (51.7%) faculty received their internet connection through W-LAN (wireless connection, local area network), followed (34.5%) by the LAN (connection).This enables them to conduct their teaching wherever there is wireless internet connectivity.

Internet connection used	N (%)
Mobile network	I (3.5%)
W-LAN (wireless connection)	15 (51.7%)
LAN (cable connection)	10 (34.5%)
Both WLAN and LAN	3 (10.3%)
Total	29 (100%)

Table 4. Shows type of internet connection used.

Table 5. Shows themes and subthemes for those who enjoyed online teaching (48.3%, n=14) in response to the question whether one enjoyed online teaching along with disadvantages and advantages of online teaching, convenient for teaching, cost effective, and safe teaching modality under the COVID-19 pandemic.

Number	Theme	Subtheme
1	Convenient for teaching	It is a convenient method<br>It is flexible<br>Effective with big numbers<br>More practical because one doesn't have to travel to teach<br>I can teach from my office without having to go to different lecture rooms for different year groups<br>It is convenient as I can continue with my teaching even when I am not physically in the country<br>Teaching can be done at a convenient time<br>It is suited for a busy clinician
2	Cost effective	It is cheaper<br>It cuts on transport cost<br>A lecture can be recorded for future us<br>Challenges are welcome
3	More effective teaching methods explored and applied	Gives room to explore other ways of teaching like simulation and videos<br>Helps facilitator simplify teaching and produce quality lecture presentations.<br>It is easier to attach videos and pictures to illustrate a point.
4	. Safer in the prevailing COVID-19 pandemic	It is safer especially in this prevailing COVID-19 pandemic.<br>A novel method.

Table 5. Shows themes and subthemes for those who enjoyed online teaching in response to the question whether one enjoyed online teaching.

Table 6. Shows the themes and subthemes for those who indicated that they did not enjoy online teaching (51.7%, n=15) in response to the question whether one enjoyed teaching online. They enumerated some of the disadvantages of online teaching such as clinical teaching and difficulty to give timeous feedback to students.

Eighty three per cent (83%, n=24) of faculty had commenced online teaching and learning with 17% (n=5) yet to commence teaching online.

Number	Theme	Sub-theme
1	Lack of physical interaction with students	I am comfortable with my students<br>It lacks interaction<br>You cannot see students as you teach so you don't know if what you deliver is effective and understood<br>I prefer physical interaction with students<br>Less interaction with students<br>I teach better with face –to- face teaching

		I do not link with 60% of my students as they depend on other students as they re-route information via other platforms like WhatsApp<br>I cannot see students as they switch off their videos
2	Frequent disruption of lecture due to network challenges	Internet connectivity not good<br>This is a whole new experience which is marred by lack of suitable internet connectivity, lack of data and resources.<br>Network challenges can be a problem<br>I often have internet problems leading to disruption in teaching
3	Lack of immediate feedback	Student facial response and engagement allows me to continuously modify my teaching<br>It is not possible to assess facial expressions to see if students understood.<br>There is lack of immediate feedback.
4	Unsuitable for practical based learning	Online learning is unsuited for practical based learning

Table 6. Shows themes and subthemes for those who indicated that they did not enjoy online teaching in response to the question whether one enjoyed teaching online.

Table 7, shows the platforms in use for online teaching. The most commonly used platform is ZOOM (62.1%, n=18).

Platform	N/%
Zoom	18/62.1%
Moodle	5/17.2%
E-Mhare	2/6.9%
Google meet	1/3.4%
Google classes	1/3.4%
Zoom and Google	1/3.4%
WhatsApp and emails	1/3.4%
Totals	29/100%

Table 7.Platforms used for online teaching

Table 8. Shows barriers to online teaching, learning and assessment and possible solutions**.** Internet connectivity, high data costs, and inability to pass on technical skills were some of the barriers to online teaching. Solutions offered included a subsidy to faculty to access data and incorporate facilities that would beam online activities in the wards.

Barrier	Possible Solutions
1.University internet connectivity may be unavailable, disrupted or broken down	Lecturers and students should be supported financially so that they secure data for efficient uninterrupted flow of lectures.

2. Non-availability of allowances for internet for lecturer	Provide data or allowance for internet to lecturers to enable smooth lecture planning and teaching off campus.
3. Unable to pass on technical skills as bedside teaching is impossible	To accommodate physical lectures in wards in turn with online teaching.
4. Data costs too high if venue of teaching is not the workplace.	To provide lecturers with internet allowance.
5. Lectures disrupt by use of the limited 40 minute zoom session and time wasted when re-logging in.	The institution to subscribe to the unlimited facility.
6.Unvavailability of internet equipment in the units	The purchase of internet equipment by the institution
Inattention by students	More training is required about the monitoring of student attendance and participation.

Table 8. Shows the barriers and possible solutions to online teaching and learning

DISCUSSIONS

The lockdowns and subsequent closure of higher education institutions are mitigation measures to the spread of the SARS-CoV-2 virus and protect the students, faculty and the community but at the same time disrupted the training of future healthcare professionals due to loss of time of learning/training and inability to continue adequate clinical training [15].The COVID-19 pandemic has caused the closure of university campuses around the world and migration of all learning, teaching, and assessment to online domains [2]. This has caused profound changes in health professions education with regards to clinical placements along with the social suffering and extensive economic hardships and strain on healthcare systems. High income countries (HIC) managed to swiftly migrate to e-learning with ease as they already had invested in digitalisation unlike the low and middle income countries (LMIC) that had minimal capacity and require extensive adjustments to achieve the abrupt switch to e-learning [1,17-22]. The e-learning technology has not been evenly dispersed throughout all nations and cultures [22].Limited institutional capacity and knowledge on online teaching inevitably made this transition more challenging for LMIC. The HIC successfully swiftly implemented a vast array of technology-enhanced learning solutions within the pre-existing structures: webinars conducted via zoom, Skype, Google Hangouts, WebX; online learning platforms; mobile applications; 3-dimensional anatomy models; and online question banks [19, 23, 24]. In some LMIC distance e-learning had not been adopted as a modality of teaching within health professions education [19]. The availability of essential infrastructures, well-trained educators, advanced technologies, efficient institutional strategies and student related barriers represent major challenges in integrating distance e-learning in health professions education in LMIC [23-26]. With the uncertainty of returning to normal life after the containment of the pandemic e-learning will remain the modality of teaching, learning and assessment in higher education.

Digital learning is a process of integrating technology-mediated synchronous and asynchronous approaches including assessments, assignments, and tutoring enabling learning without any time and location restrictions 15, 27, 28]. Digital learning has a few components including digital teaching materials, digital tools, digital delivery, and autonomous learning. Health professions education has adopted digital learning with through virtual course, simulation software and teleconferencing and are struggling to come up with suitable virtual teaching and learning for the clinical component of their training that requires the traditional patient contact in the hospital/clinic set up.

This is in general agreement that online learning is a good option for acquiring theoretical knowledge since it is not possible to hold face-to-face learning under the COVID-19 pandemic. Efficacy of online learning has long been acknowledged [29 -34]. Hugenholtzel found that e-learning is just as effective in enhancing knowledge as lecture-based learning [31]. A study indicated that online learning was less effective in building skills and knowledge mainly due to the difficulty of interacting with peers and faculty along with the acquisition of skills which are mostly better acquired through in-person interaction [7]. However, literature indicates that high level of satisfaction are related to well -structured and organised e-courses which also have a greater impact on knowledge accumulation and student performance compared with traditional learning [7]. Systematic reviews

have reported that blended learning has better effects on knowledge outcomes compared to traditional learning [33, 36].

Blended learning has been adopted by our institution with small groups of students attending clinical settings and in-person lectures intermixed with online learning.

In the current study the majority of faculty (65.5%) had received training in online teaching, learning and assessment. In line with other studies in the literature the technological skills to provide online courses increases the educational value of faculty. [37]. There is need to continue to support faculty as they implement e-learning.

Twenty nine (29) lecturers participated in the study: 62.1% (n=18) were males and 37.9% were females. The majority of the respondents were in the lecturer (37.9%) and senior lecturer grade (34.5%). This could reflect a relatively low experience in teaching and possibly age hence their perception of e-learning is likely to be positive based on their high technology capacity. This could also explain their enjoyment of online teaching, learning and assessment. Fischer et al. stated that older staff with long traditional teaching experience usually have limited interaction with technology and lacking the development of their necessary skills [38,39].

The most commonly used device for the delivery of teaching was the laptop (82.7%) followed by the personal computer (10.3%). The institution would then be expected to assist faculty with replacement of laptops as support for the continued online teaching, learning and assessment.

Faculty most commonly used W-LAN (51.7%) followed by the LAN (34.5%).The institution would be expected to improve the internet connectivity at the institution and also assist faculty with data when they are away from the office so that they could continue with online teaching wherever they would be especially with the lockdown measures.

In agreement with other studies in the literature the majority of respondents (93.1%) believed that there were benefits in online teaching [37, 38]. Literature indicates that some of the factors among faculty that influence the level of satisfaction of online teaching are self-gratification, intellectual challenge, interest in using technology, and associated professional development opportunities [5,40]. This study found that the benefits of e-learning were: convenience for teaching, cost effectiveness, effective teaching approach and a safer teaching approach under the COVID-19 pandemic. Most institutions were using blended learning before the advent of the COVID-19 pandemic and did find the switch to completely online overwhelming.

Six point nine percent (6.9%) did not agree that there were any additional benefits compared with face-to-face teaching and that immediate feedback is curtailed, lack of physical interaction with students, network challenges affecting lecture delivery and suitability for practical-based learning, and negative impact on hands on training or skills transfer. They also implored for the need to hold regular training courses on online teaching and learning. This is in agreement with other studies in the literature that showed that some faculty were not fully comfortable with e-learning as a teaching tool and attributed this to factors such as technological challenges, difficulty in interactions and discussions with students, lack of adequate internet connectivity, students access to internet was difficult and expensive, and personal learning preference [37,38].There is need to cultivate a culture of change as institutions transition to online teaching, learning and assessment as this will reduce resistance. Advanced planning and infrastructure are important for successful implementation of e-learning [41]. There is also need to modulate the traditional methods of teaching, learning and assessment for e-learning.

Ninety three per cent (93.0%; n=27) reported that they believed in the benefits of online teaching of students with 7 %(n=2) indicating that there were no benefits to students accruing from online teaching. The majority of faculty agreed that online teaching enhanced their teaching capacity while those who did not believe in the enhancement from online teaching felt that it added no value to the learning of the students. They indicated the need for further training and adequate appropriate tools and reliable internet connectivity.

The majority (83%; n=24) are already participating in online teaching with zoom being the most common platform used (62.1%) with social media and email being used to communicate with students. Other platforms were also used: Google meet, Google lessons, e-Mhare. In our faculty both synchronous (live or in real-time) and asynchronous (recorded or self-paced) are used through the University's learning management system (along with Zoom or Microsoft Teams). Synchronous e-learning is offered in the form of interactive teaching and clinical case discussions in small and large group formats [37] and asynchronous e-learning includes

preparation of course materials for students in advances to be accessed later (recorded lectures, supportive videos, external links for recommended websites, and electronic books [37,41].

Literature indicates that there are many significant barriers to the adoption and implementation of e-learning by medical schools [5, 10]. These barriers include technology/infrastructure barriers, institutional/educators' and student barriers [19]. In agreement with other studies from LMIC lack of infrastructure, advanced technology, internet access, and poor quality of internet services (internet band width and connectivity limitations), limited technical support, unfamiliarity with e-learning systems were mentioned as barriers to the transition to virtual teaching/training and learning [15,19,20] : unavailability of university internet connectivity (unavailable, disrupted, broken down, interrupted power supply), absence of financial allowances to pay for internet service data (high cost) , inattention by students, inability to perform skills transfer , disturbances from the home environment (working at home due to lockdown measures). The disturbances at home are similar to those in other studies which showed that the learning environment at home posed the greatest challenge while technological literacy and competency was the least of their challenges [34].Low household income in LIMC makes it difficult for one to access the necessary equipment for online learning, along with poor power supply, poor internet connectivity, sociocultural dynamics as gender roles, educators' and learners' competence related to online teaching.

Blended teaching and learning, combining online and face-to-face teaching, balances benefits and drawbacks of online and face-to-face teaching [30]. Distance online course can prepare students as well as face-to-face. However, performance in online learning is reduced due to the COVID-19 driven social distancing and physical isolation as activities such as workshops, laboratories, and clinical rotations have had to be suspended [30].

The teaching environment poses challenges met by faculty inclusive of issues such as distractions at home (e.g., visitors, noise, and home chores) and limited space and facilities [34]. Poor internet connectivity, poor picture quality of videos and poor performing platforms were noted as possible barriers [25].Students and faculty need continuing training on online teaching and learning. A needs assessment should be performed among the students, health professions educators and the institution so that the identified needs and quality control be implemented at commencement of online teaching [35, 36, 42]. Poor internet connectivity, poor picture quality of videos and poor performing platforms were noted as barriers in this study as was noted in other studies [25].The needs assessment would help in the development of appropriate policies and a supportive culture.

Conclusions

The impact of the emergency embarking on online learning is not yet known. Early stages yet to assess the role of the academics on the future of e-learning in global health professions education. These findings can be used to improve health professions faculty approach to online teaching and learning as the global COVID-19 pandemic appears to be with us for a long time to come.

Institutions should improve the online teaching and learning infrastructures: learning management system (webinar facilities (zoom etc.) online learning platforms, mobile applications, Skype, Google Hangouts, training of both students and faculty on online teaching and learning, simulation/skills, laboratories, develop online curriculum, develop online examinations, telehealth, online video libraries. The developments should be embarked upon in line with their unique challenges of their faculty and the institutional existing online infrastructures. Continuing training and support on e-learning for faculty is important for successful acceptance and implementation of e-learning.

Methods of introducing and supporting health professions faculty transitioning to new teaching and learning circumstances and environments should provide contextualised both online and in-person strategies. Consideration of the negative and positive aspects of e-learning should allow for the development of a blended learning approach , flipped class, team-based learning approach that integrates aspects of face-to-face learning and e-learning to achieve the intended learning outcomes especially in practical and clinical training.

REFERENCES:

1. WHO (2020).WHO Director- General's Opening Remarks at the Media Briefing on COVID-19- 11 March 2020. Available online at: https://www.who.int/director-general/speeches/detail/who-director general-s-opening-remarks-at-the-media-briefinh-on-covid-19- 11-March-2020. (Accessed September 30:2021.

2. Watermeyer R, Crick T, Knight C, Goodall J. COVID-19 and digital disruption in UK universities: afflictions and affordances of emergency online migration. Higher Education.2021; 81:623-641.

3. The Lancet. Editorial 2020: Redefining vulnerability in the era of COVID-19.https://doi.org/10.1016/50140-6736 (20)30757-v

4. UNESCO COVID-19. Educational disruption and response 2020. (Available from https://en.unesco.org/themes/education-emergenices/coronavirus-school-closure.

5. Rad FA, Otaki F, Baqain Z, et al. Rapid transition to distance learning due to COVID-19: Perceptions of postgraduate dental learners and instructors. PLOS ONE.2021.https://doi.org/10.137/journal.pone.0246584.

6. Kernohan D. Which universities are moving to remote teaching? WONKHE 2020:https://wonke.com/blogs/which-universities-are-moving-to-remote-teaching/

7. Al Qhtani A, Al Swedan N, Almulhim A. et al. Online versus classroom teaching for medical students during COVID-19: measuring effectiveness and satisfaction. BMC Med Educ.2021; 21: 452-8.

8. Crawford J, Butler-Henderson K, Rudolph J, et al. M. COVID-19: 20 Countries' higher education intra-period digital pedagogy responses. JAppl.Learn.Teach2020;3:1 340-389.https;//journal.sfu.ca/jalt/index.php/jalt/index1-

9. Adman M, Anwar K. Online learning amid the COVID-19 pandemic: students 'perspectives. Journal of Pedagogical Sociology and Psychology.2020; 2: 45-51.

10. Kaur G. Digital life: Boon or bane in teaching sector on COVID-19.CLIO an Annual Interdisciplinary Journal of History.2020; 6: 416-427.

11. Schlenz MA, Schmidt A, Wostmann B et al.Students' and lecturers' perspectives on the implementation of online learning in dental education due to SARS-CoV-2 (COVID-19): a cross-sectional study. BMC Medical Educ2020; 20:354-361.

12. Ebner M, Schon S, Braun C et al. COVID-19 pandemic as E-learning boost? Chronological development and effects at an Austrian University against the background of the concept of "E-learning Readiness". Future Internet2020; 12:354-371.

13. Tabatabai S. Simulations and virtual learning supporting clinical education during the COVID-19 pandemic. Adv Medic Educ and Pract2020; 11:513-516.

14. Ahmed H, Alf M, Elghazaly H.COVID-19 and medical education.Lancet.2020; 20:777-778.

15. Kiguli-Malwadde E. COVID-19 and its impact on health professions education in Africa. Afr Health.2020; 13:12-13.

16. Papapanou M, Routsi E, Tsamkis K et al. Medical education challenges and

innovations during COVID-19 pandemic. Postgrad Med J.doi:1136/postgrad 2021-140032.

17. Doherty J.Education and training update: e-learning in clinical teaching. Brit J Hosp Med. 2010; 71:44-47.

18. Wang Y, Rongbin Y, Liu Y et al.Qian W (2021). Students' and Teachers' perspective on the implementation of medical education in China: a qualitative study. Adv Med Educ Pract 12; 895-902.

19. Al-Balas M, Al-Balas H I, Jaber HM et.al. Distance learning in clinical medical education amid COVID-19 pandemic in Jordan: current situation, challenges, and perspectives 2020; 20: 341-8.

20. Gismalla MDA, Mohamed MS, Ibrahim OSO et. Al. Medical students' perception towards e- learning during COVID-19 pandemic in a high burden developing country 2021; 21:3 77-13.

21. Olum R, Alulinda L, Kigozi E et.al. Medical education and e-learning during the COVID-19

pandemic: awareness, attitudes, preferences, and barriers among undergraduate medicine and nursing students at Makerere University, Uganda.2020; 7:1-9.

22. Li W, Gillies R, He M et al. Barriers and facilitators to online medical and nursing education during the COVID-19 pandemic: perspectives from international students from low- and middle- income countries and their teaching staff. Hum Resourc Health 2021;19:64-78.https://doi.org/10.1186/3129960-021-00609-9

23. Panahl P, Borna F.Distance learning challenges: challenges, new solution 2014 37th International Convention on Information and Communication Technology .Opatija: Electronics and Microelectronics (MIPRO); 2014. P653-6. https://doi.org/10.1109/MIPRO.20146859648.20.

24. Dawidziuk A, Kawkka M, Szyszka B et al. Global access to technology-enhanced medical education during the COVID-19 pandemic: the role of students in narrowing the gap. 2021; 9:S1-S5.

25. Di Giano P, Di Paolo C. COVID-19 and dental distance-based education: students' perception in an Italian University. BMC Med Educ.2021; 21:414-9.

26. Sigdel S, Ozaki A, Dhakal R, et al. Medical education in Nepal: Impact and challenges of the COVID-19 pandemic. Acad Med 2021; 96: 340-342.

27. Xin LJ, Hathim AAH, Yi NJ et al. Digital learning in medical education: comparing experiences of Malaysian and Japanese students. BMC Med Educ 2021; 21: 418-9.

28. Kiesewetter J, Sailer M, Jung VM et al. learning clinical reasoning how virtual patient case format and prior knowledge interact. BMC Medl Educ.2020;20;73.https://doi.org/10.1186/s12909-020-1987-y.

29. Dost S, Hossain A, Shehab M et al. Perceptions of medical students towards online teaching during the COVID-19 pandemic: a national cross-sectional survey of 2721 UK medical students. BMJ Open 2020;10:e042378.doi:10.1136/bmj/open-2020-042378.

30. Rossettini G, Geri T, Turolla A et al. Online teaching in physiotherapy education during COVID-19 pandemic in Italy: a retrospective case-control study on students' satisfaction and performance. BMC Med Educ 21;2021;21:456-12.

31.Hugenholtz NI, de Groun EM,Smits PB et al.Effectiveness of e-learning in continuing Vallee A, BlacherJ,Carlou A. Sorbets E. Blended learning compared to traditional learning in medical education: systematic review and meta- analysis.J Med Internet Res.2020;22(8)e16504.http://doi.org/10.2196/16504.PMID:32773378;PMCID:PMC7445617.

32. Wilcha RJ. Effectiveness of virtual medical teaching during the COVID-19 crisis. Systematic Review.JMIR MedEdu.2020; 6(2):e20963.https://doi.org/10.21963.PMID:33106227:PMCID:PMC7682786.

33. Baherimoghadam T, Hamedani S, Mehrabi M et al. The effect of learning style and general self-efficacy on satisfaction of e-learning in dental students. BMC Med Educ 2021; 21: 463-9.

34. Barrot JS, Linares II, Del Rosario LS. Students' online learning challenges during the pandemic and how they cope with them: the case of the Phillippines.Educ Infor Tech.https://doi.org/10.1007/s10639-021-10589-x.

35. Ardekani A, Hosseini SA, Tabari P et al. Student support systems for undergraduate medical students during the COVID-19 pandemic: a systematic narrative review of the literature. BMC Med Edu. 2021; 21:352-64.

36. Ashoka B, Ong SY, Tay KH et al.Coordinated responses of academic medical centres to pandemics: sustaining medical education during the COVID-19 pandemic. Med Teacher. https://doi.org/10.1080/01422159X.2020.1757634.

37. Zalat MM, Hamed MS, Bolbot SA. The experiences, challenges, and acceptance of e-learning as a tool for teaching during the COVID-19 pandemic among university medical staff. 2021. PLoS ONE 16(3):e0248758.https://doi.org/10.1371/journal.pone0248758.

38. Hong KS, Lai KW, Holton D (2004). Students. Satisfaction and perceived learning with a web- based course. J Educ Tech Soc 2004; 6:1-12.

39. Fischer SH, David D, Crotty BH et al. (2014). Acceptance and use of health information technology by community-dwelling elders.Int J Med Infor 2014; 83: 624-35.

40. Bolliger DU, Wasilik O. Factors influencing faculty satisfaction with online teaching and learning in higher education. Dist Educ 2009; 30: 103-16.

41. Almuwals A, Alqabbani S, Benajiba N et al. (2021). An emergency shift to e-learning in health professions education: a comparative study of perspectives between students and institution. Int J Learn Teach Educ Res2021; 20: 16-37.

42. Mishra L, Gupta T, Shree A .Online teaching-learning in higher education during lockdown period of COVID-19 pandemic 2020.Int Journal Edu Res Open. https://doi/10.1016/j.ljedro.2020.10012.

Manuscript Click here to access/download;Manuscript;Manuscript .docx

PLOS ONE

Health professions faculty's perceptions of online teaching and learning during the COVID-19 pandemic

--Manuscript Draft--

Manuscript Number:	PONE-D-21-33695
Article Type:	Research Article
Full Title:	Health professions faculty's perceptions of online teaching and learning during the COVID-19 pandemic
Short Title:	Health professions educators' perceptions online teaching under COVID-19 pandemic
Corresponding Author:	Midion Mapfumo Chidzonga, BDS FFDRCSI<br>Professor<br>Harare, ZIMBABWE
Keywords:	COVID-19 pandemic; higher education; online teaching, learning and assessment, health professions faculty
Abstract:	The global societal impact of the COVID-19 pandemic is incalculable with profound social suffering, deep economic hardships and enforced closure of schools, businesses, and higher learning institutions through the imposition of lockdown and social distancing in mitigation of the spread of the SARS-Cov-2 infection. Institutions have had to hastily migrate teaching, learning and assessment to online domains, at times with ill-prepared academics, students and institutions and with unwelcome and disorienting consequences. Our study surveyed perspectives of faculty at the University of Zimbabwe Faculty of Medicine and Health Sciences (UZFMHS) towards the hastily adopted online teaching, learning and assessment implemented in response to the mitigation of the COVID-19 pandemic. Twenty-nine (29) faculty from the major disciplines and career hierarchy participated in the study There were mixed responses regarding the use of this modality for teaching, learning and assessment: training before online teaching, learning and assessment, advantages and disadvantages, cost effectiveness, effectiveness for teaching, learning and assessment, effect on student feedback, disruptions from internet connectivity issues, interaction with students, suitability for practical training, and barriers to online teaching, learning and assessment. These results would enable the UZFMHS develop institutional and personalised approaches that would enable execution of online teaching, learning and assessment under the current and post COVID-19 pandemic.
Order of Authors:	Midion Mapfumo Chidzonga, BDS FFDRCSI
	Clara Opha Haruzivishe, PhD
	Judith Rukweza, PhD
	Vasco Chikasha, PhD
Additional Information:	
**Question**	**Response**
**Financial Disclosure**<br><br>Enter a financial disclosure statement that describes the sources of funding for the work included in this submission. Review the submission guidelines for detailed requirements. View published research articles from *PLOS ONE* for specific examples.<br><br>This statement is required for submission and **will appear in the published article** if	1.All authors were under the same funding: initials are MMC, COH, JR, VC<br>2.www.norad.no<br>3. Funded by the Norwegian Programme for Capacity Development in Higher Education and Research Development<br>(NORHED)<br>4. NO.The funders had no role in study design, data collection and analysis, decision to publish, or preparation of the manuscript

Powered by Editorial Manager® and ProduXion Manager® from Aries Systems Corporation

the submission is accepted. Please make sure it is accurate.<br><br>**Unfunded studies**<br>Enter: *The author(s) received no specific funding for this work.*<br><br>**Funded studies**<br>Enter a statement with the following details:<br>• Initials of the authors who received each award<br>• Grant numbers awarded to each author<br>• The full name of each funder<br>• URL of each funder website<br>• Did the sponsors or funders play any role in the study design, data collection and analysis, decision to publish, or preparation of the manuscript?<br>• **NO** - Include this sentence at the end of your statement: *The funders had no role in study design, data collection and analysis, decision to publish, or preparation of the manuscript.*<br>• **YES** - Specify the role(s) played.<br><br>* typeset	
**Competing Interests**<br><br>Use the instructions below to enter a competing interest statement for this submission. On behalf of all authors, disclose any competing interests that could be perceived to bias this work—acknowledging all financial support and any other relevant financial or non-financial competing interests.<br><br>This statement is required for submission and **will appear in the published article** if the submission is accepted. Please make sure it is accurate and that any funding sources listed in your Funding Information later in the submission form are also declared in your Financial Disclosure statement.<br><br>View published research articles from *PLOS ONE* for specific examples.	NO. The authors have declared that no competing interests exist.

**NO authors have competing interests**<br><br>Enter: *The authors have declared that no competing interests exist.*<br><br>**Authors with competing interests**<br><br>Enter competing interest details beginning with this statement:<br><br>*I have read the journal's policy and the authors of this manuscript have the following competing interests: [insert competing interests here]*<br><br>* typeset	
**Ethics Statement**<br><br>Enter an ethics statement for this submission. This statement is required if the study involved:<br><br>• Human participants<br>• Human specimens or tissue<br>• Vertebrate animals or cephalopods<br>• Vertebrate embryos or tissues<br>• Field research<br><br>Write "N/A" if the submission does not require an ethics statement.<br><br>General guidance is provided below. Consult the submission guidelines for detailed instructions. **Make sure that all information entered here is included in the Methods section of the manuscript.**	Conflicts of interest/Competing interest: None<br>Availability of data and material (data transparency): Available<br>Code availability (software application or custom code): Not applicable<br>Authors' contributions (optional)<br>Ethics approval: Permission to carry out the study at the University of Zimbabwe Faculty of Medicine and Health Sciences (UZFMHS) was granted by the Dean of the UZFMHS. Ethical clearance to carry out the study was obtained from the local ethical review board, Joint Research Ethics Committee of the UZFMHS and Parirenyatwa Group of Hospital (JREC) and the Medical Research Council of Zimbabwe (MRCZ). Informed consent was obtained from the participants before participation in the study. No risks to the participants were envisaged during the study process. Benefits to the participants may accrue through their perspectives being used to enrich the implementation of online teaching, learning and assessment.<br>Participation was voluntary and participants were requested to read the consent form along with the information sheet and only proceed with filling in the form if they were agreeable with the information given on the consent form and the information sheet.<br>Consent to participate: Participation was voluntary and participants were requested to read the consent form along with the information sheet and only proceed with filling in the form if they were agreeable with the information given on the consent form and the information sheet.<br>Consent for publication: Not applicable<br>Text formatting: 10-point Times Roman<br><br>Acknowledgements: Many thanks to the NORHED for funding this project.

Powered by Editorial Manager® and ProduXion Manager® from Aries Systems Corporation

**Format for specific study types**<br><br>**Human Subject Research (involving human participants and/or tissue)**<br>• Give the name of the institutional review board or ethics committee that approved the study<br>• Include the approval number and/or a statement indicating approval of this research<br>• Indicate the form of consent obtained (written/oral) or the reason that consent was not obtained (e.g. the data were analyzed anonymously)<br><br>**Animal Research (involving vertebrate animals, embryos or tissues)**<br>• Provide the name of the Institutional Animal Care and Use Committee (IACUC) or other relevant ethics board that reviewed the study protocol, and indicate whether they approved this research or granted a formal waiver of ethical approval<br>• Include an approval number if one was obtained<br>• If the study involved *non-human primates*, add *additional details* about animal welfare and steps taken to ameliorate suffering<br>• If anesthesia, euthanasia, or any kind of animal sacrifice is part of the study, include briefly which substances and/or methods were applied<br><br>**Field Research**<br><br>Include the following details if this study involves the collection of plant, animal, or other materials from a natural setting:<br>• Field permit number<br>• Name of the institution or relevant body that granted permission	
**Data Availability**<br><br>Authors are required to make all data underlying the findings described fully available, without restriction, and from the time of publication. PLOS allows rare exceptions to address legal and ethical concerns. See the PLOS Data Policy and FAQ for detailed information.	Yes - all data are fully available without restriction

Powered by Editorial Manager® and ProduXion Manager® from Aries Systems Corporation

A Data Availability Statement describing where the data can be found is required at submission. Your answers to this question constitute the Data Availability Statement and **will be published in the article**, if accepted.<br><br>**Important:** Stating ‘data available on request from the author’ is not sufficient. If your data are only available upon request, select ‘No’ for the first question and explain your exceptional situation in the text box.<br><br>Do the authors confirm that all data underlying the findings described in their manuscript are fully available without restriction?	
**Describe where the data may be found in full sentences. If you are copying our sample text, replace any instances of XXX with the appropriate details.**<br><br>• If the data are **held or will be held in a public repository**, include URLs, accession numbers or DOIs. If this information will only be available after acceptance, indicate this by ticking the box below. For example: *All XXX files are available from the XXX database (accession number(s) XXX, XXX.*).<br>• If the data are all contained **within the manuscript and/or Supporting Information files**, enter the following: *All relevant data are within the manuscript and its Supporting Information files.*<br>• If neither of these applies but you are able to provide **details of access elsewhere**, with or without limitations, please do so. For example:<br><br>*Data cannot be shared publicly because of [XXX]. Data are available from the XXX Institutional Data Access / Ethics Committee (contact via XXX) for researchers who meet the criteria for access to confidential data.*<br><br>*The data underlying the results presented in the study are available from (include the name of the third party*	Not applicable

and contact information or URL).<br>• This text is appropriate if the data are owned by a third party and authors do not have permission to share the data.<br><br>* typeset	
Additional data availability information:	Tick here if your circumstances are not covered by the questions above and you need the journal's help to make your data available.

Click here to access/download;Manuscript;Health Professions COVID-19.docx

TITLE PAGE

Title: Health professions faculty's perceptions of online teaching and learning during the COVID-19 pandemic

1. Midion Mapfumo CHIDZONGA

Department of Oral Health

Faculty of Medicine and Health Sciences

University of Zimbabwe

HARARE

ZIMBABWE

mtmchidzonga@yahoo.com

ORCID http://orcid.org/0000-0002-4574-1158

2. Clara HARUZIVISHE

Department of Primary Health Care

Unit of Nursing Sciences

Faculty of Medicine and Health Sciences

University of Zimbabwe

HARARE

ZIMBABWE

claraopha@gmail.com

3. Vasco CHIKWASHA

Unit of Global and Public Health

Department of Primary Health Care

Faculty of Medicine and Health Sciences

University of Zimbabwe

HARARE

ZIMBABWE

vchikasha@gmail.com

4. Judith RUKWEZA

Nursing Sciences

Department Primary Health Care

Faculty of Medicine and Health Sciences

University of Zimbabwe

HARARE

ZIMBABWE

rukwezajudith@gmail.com

Corresponding Author: Midion Mapfumo Chidzonga

mtmchidzonga@yahoo.com

ABSTRACT

The global societal impact of the COVID-19 pandemic is incalculable with profound social suffering, deep economic hardships and enforced closure of schools, businesses, and higher learning institutions through the imposition of lockdown and social distancing in mitigation of the spread of the SARS-Cov-2 infection. Institutions have had to hastily migrate teaching, learning and assessment to online domains, at times with ill-prepared academics, students and institutions and with unwelcome and disorienting consequences. Our study surveyed perspectives of faculty at the University of Zimbabwe Faculty of Medicine and Health Sciences (UZFMHS) towards the hastily adopted online teaching, learning and assessment implemented in response to the mitigation of the COVID-19 pandemic. Twenty-nine (29) faculty from the major disciplines and career hierarchy participated in the study. There were mixed responses regarding the use of this modality for teaching, learning and assessment: training before online teaching, learning and assessment, advantages and disadvantages, cost effectiveness, effectiveness for teaching, learning and assessment, effect on student feedback, disruptions from internet connectivity issues, interaction with students, suitability for practical training, and barriers to online teaching, learning and assessment. These results would enable the UZFMHS develop institutional and personalised approaches that would enable execution of online teaching, learning and assessment under the current and post COVID-19 pandemic.

Keywords: COVID-19 pandemic; higher education; online teaching, learning and assessment, health professions faculty.

DECLARATIONS

Funding: Norwegian Programme for Capacity Development in Higher Education and Research Development (NORHED)

Conflicts of interest/Competing interest: Not applicable

Availability of data and material (data transparency): Available

Code availability (software application or custom code): Not applicable

Authors' contributions (optional)

Ethics approval: Permission to carry out the study at the University of Zimbabwe Faculty of Medicine and Health Sciences (UZFMHS) was granted by the Dean of the UZFMHS. Ethical clearance to carry out the study was obtained from the local ethical review board, Joint Research Ethics Committee of the UZFMHS and Parirenyatwa Group of Hospital (JREC) and the Medical Research Council of Zimbabwe (MRCZ). Informed consent was obtained from the participants before participation in the study. No risks to the participants were envisaged during the study process. Benefits to the participants may accrue through their perspectives being used to enrich the implementation of online teaching, learning and assessment.

Participation was voluntary and participants were requested to read the consent form along with the information sheet and only proceed with filling in the form if they were agreeable with the information given on the consent form and the information sheet.

Consent to participate: Participation was voluntary and participants were requested to read the consent form along with the information sheet and only proceed with filling in the form if they were agreeable with the information given on the consent form and the information sheet.

Consent for publication: Not applicable

Text formatting: 10-point Times Roman

Automatic page numbering

Acknowledgements: Many thanks to the NORHED for funding this project.

INTRODUCTION: The World Health Organisation (WHO) declared COVID-19 a global public health emergency of international concern on 30th January 2020 and subsequently declared it a pandemic on 11th of March [1, 2]. The societal impact of COVID-19 is incalculable as the pandemic continues to cause profound social suffering and deep economic hardships especially for society's most vulnerable and less fortunate [1,2,3]. Many aspects of life have been seriously disrupted by the COVID-19 pandemic. The lockdown measures, in a bid to mitigate the spread of the SARS-Cov-2 infection, have led to enforced closure of schools, colleges, and universities, but without cessation of all learning, teaching and assessment although many forms of assessment have had to be suspended [2,4]. Most institutions of higher learning had to hastily migrate to the online domains with some of them with poor online infrastructures as well as ill-prepared academics and students [5, 6, and 7]. The new experience has been unwelcoming and disorienting to some educators, and students alike. The pandemic has also exposed deficiencies in the use of technology in higher education, which has long been debated, and is now overdue [2]. The University of Zimbabwe Faculty of Medicine and Health Sciences (UZFMHS) now has to embrace this change hastily. This new challenge along with the attendant obstacles has to be faced by many higher education institutions [8].There is no indication when the pandemic will be contained and as such educational institutions across the globe have now opted to use the available technical resources to create online teaching, learning and assessment material for students of all academic levels [9,10].The pandemic has thus created a forced digitalisation change, which was long overdue, and an opportunity to accelerate digital transformation, in health professions education [11,12].This is a positive move post COVID-19 pandemic. Pre- clinical health professions education can be taught through webinars. Unfortunately, online teaching, learning, and assessment is most useful for theoretical content but innovative ways have had to be developed to enable clinical training and assessment [13,14,15,16].It is known that formative and high-stakes examinations could be carried out by modifying the current assessment processes such as multiple choice questions, short-answer questions can be done online at the same time viva voce, teleconferencing between candidate and examiners; use of simulation/ objective structured clinical examination; modify patient contact stations to simulated patients; task-trainers and hybrid simulations; patient requiring components; use of personal protective equipment (PPE) at each station; examiner view the examination process remotely .Clinical training depends heavily on hands-on- patient contact to enable clinical skills acquisition, practical work in the laboratories which would require advanced technology which is currently unavailable in most low and middle income countries (LMICs). However, collaboration with high income countries (HIC) may alleviate this situation. Innovative approaches are being put in place to enable continued clinical training: vaccination of all students and faculty; provision of adequate PPE to students, faculty and patients; good hand hygiene; mask wearing; face shields; social distancing; full-body PPE(long-sleeved disposable gown, eye protection, gloves) for aerosol producing procedures; and small numbers of students at a time. For non-aerosol producing procedures, minimum PPE is provided (disposable apron, facemask, gloves and eye protection is provided. This ensures that the safety of patients, students and staff is not compromised.

Health professions students need to be exposed to patients in an authentic environment, an apprenticeship type of learning. This is usually in hospital wards, outpatient clinics and simulated teaching laboratories. Under the current COVID-19 pandemic live broadcasts, films or virtual case presentations, and surgical procedures might be used to complement the limited access to patients. Faculty are expected to increase their digital competence so as to come up with innovative virtual teaching, learning, and assessment tools.

The COVID-19 pandemic has curtailed clinical placements as well as elective services offered in clinical services. However, there is need to continue to adequately train health professionals to run the healthcare system. Student clinical placement has to continue while observing safe supervision of students, adequate provision of PPE, social distancing, washing and sanitising hands. Various learning management systems are available: Moodle, Massive Open Online Courses. Several tools are also available that allow for video communications, video and audio conferencing, chats, and webinars: Skype, Zoom, and Google Hangouts Meet, Google school [13].It is possible to set up real-time classroom experience that simulates patient encounters using live HD-quality mobile or fixed-cameras that ensures that all distance learners can collaborate at an equivalent baseline [14].Current virtual learning management systems offer access to educational content from anywhere, synchronously or asynchronously with interactive simulation learning [13].Unfortunately the theoretical knowledge has the challenge of not being able to be transferred to patient management. The way forward would be to introduce interactive simulation learning (computerised simulation education) which unfortunately need massive infrastructure ranging from clinical simulation management software and hardware, design and planning tools, file backup, cloud-based eLearning, and expert teams to provide support to counsellor education, case developers, and virtual patient training [13]. This study at the UZFMHS surveyed the faculty's perspectives towards online teaching, learning and assessment. The information gathered would enable development of online approaches tailor made based the perceptions of faculty.

METHODOLOGY

SETTING: The study was conducted at the UZFMHS which provides health professions education and training to various healthcare cadres.

STUDY DESIGN: Mixed method design.

ETHICAL CONSIDERATIONS: Permission to carry out the study was obtained from the Dean of the UZFMHS. Ethical clearance was obtained from the local ethical review board, Joint Research Ethics Committee of the UZFMHS and Parirenyatwa Group of Hospital (JREC) and the Medical Research Council of Zimbabwe (MRCZ). Informed consent was obtained from the participants. Participation was voluntary. There were no risks to the participants. Participation was voluntary and participants were requested to read the consent form and only proceed with filling in the form if they were agreeable with the information given on the consent form. Benefits to the participants may accrue should their perspectives be used to enrich the development of online teaching, learning and assessment.

STUDY PARTICIPANTS: The participants were faculty of medicine, dentistry, nursing, physiotherapy, occupational therapy, pharmacy, health promotion and education, radiographers and medical laboratory scientists.

SAMPLING: A convenient sample of 100 faculty from the above disciplines was selected from those who were accessible on campus during the time of data collection. Only 29 faculty responded.

STUDY INSTRUMENTS: An online questionnaire was created in Google Forms and emailed to faculty. The questionnaire captured demographics, academic grade, number of years in teaching, computer literacy, device used when delivering teaching type of internet connections used, whether one received training on online teaching and learning, benefits of online learning to students, role of online learning in enhancing teaching, belief in online teaching, cost effectiveness, convenience for teaching, disadvantages and advantages of online education, barriers to online teaching, and online teaching practice, and platforms used in teaching. The tool used to collect data was piloted on 5 faculty who were excluded from the main study. Following this pilot some items were removed and only relevant items were left: three 3 questions relating to demographic data and 13 questions relating to online teaching and learning were finally used for the study.

DATA ANALYSIS: Data collected through the online survey was exported to STATA 15.1 statistical software for cleaning, analysis, calculation frequencies and percentages for categorical data. Qualitative data is presented as themes and subthemes. Frequency responses to "YES" and "NO" items are presented in the tables.

RESULTS:

Twenty-nine (29) out of a hundred (100) faculty responded, 62.1% (n=18) males and 37.9% (n=11) females. The teaching experience of faculty ranged from 2 years to 44 years. A total of 19 (65.5%) had facilitated online teaching and 10 (34.5 %) had not.

On belief in online teaching, Table 1, shows responses to "Yes and "No" questions regarding online teaching, learning and assessment with 65.5% of the respondents indicating that they had received training on online teaching, learning and assessment indicative of a possible positive participation in online teaching. However, 51.7% did not enjoy teaching online with 52.0% believing that online teaching does not enhance their teaching capacity.

Table 1. Shows answers to questions regarding online teaching and learning

QUESTIONS	YES		NO	
	N	%	N	%
Have you received training on online teaching and learning?	19	65.5	10	34.5
Do you enjoy online teaching and learning?	14	48.3	15	51.7
Do you believe in benefits of online learning to students?	27	93.1	2	6.9
Do you believe that online learning enhances your teaching capacity	14	48.3	15	15.7

Those who did not believe that there were benefits in online teaching supported their views citing that there were

no additional benefits when compared to physical contacts with students and that students were less engaged and feedback is curtailed. They also pointed out the need for proper training on online teaching, requisite tools and internet access for both students and faculty as well as negative impact on hands on courses or skills transfer.

Table 2 shows that the majority of the faculty respondents are in the lecturer and senior lecturer grade. This is a positive situation as this group is likely to be in the age group that is technology savvy and should be able to navigate the education technology with ease.

Table 2. Shows academic grade of faculty.

Grade	N	%
Professor	8	(27.6
Associate Professor	0	(0.0
Senior Lecturer	10	(34.5
Lecturer	11	(37.9
TOTAL	29	100

Table 3. Shows that the majority of faculty, 85.7%, deliver their lectures using laptops which means they could deliver their teaching at any location where there is internet connectivity. One person did not specify what device they used.

Table3. Shows devices for delivering teaching.

Device	N	%
Smartphone	0	0
Tablet	1	3.6
Laptop	24	85.7
Personal Computer	3	10.7
Total	28	100

Table 4. Shows that most (51.7%) faculty received their internet connection through W-LAN (wireless connection, local area network), followed (34.5%) by the LAN (connection). This enables them to conduct their teaching wherever there is wireless internet connectivity.

Table 4. Shows type of internet connection used.

Internet connection used	N	%
Mobile network	I	3.5
W-LAN (wireless connection)	15	51.7
LAN (cable connection)	10	34.5
Both WLAN and LAN	3	10.3
Total	29	100

Table 5. Shows themes and subthemes for those who enjoyed online teaching (48.3%, n=14) in response to the question whether one enjoyed online teaching along with disadvantages and advantages of online teaching, convenient for teaching, cost effective, and safe teaching modality under the COVID-19 pandemic.

Table 5. Shows themes and subthemes in response to the question whether one enjoyed online teaching

Number	Theme	Subtheme
1	Convenient for teaching	It is a convenient method<br>It is flexible<br>Effective with big numbers<br>More practical because one doesn't have to travel to teach<br>I can teach from my office without having to go to different lecture rooms for different year groups<br>It is convenient as I can continue with my teaching even when I am not physically in the country<br>Teaching can be done at a convenient time<br>It is suited for a busy clinician
2	Cost effective	It is cheaper<br>It cuts on transport cost<br>A lecture can be recorded for future us<br>Challenges are welcome
3	More effective teaching methods explored and applied	Gives room to explore other ways of teaching like simulation and videos<br>Helps facilitator simplify teaching and produce quality lecture presentations.<br>It is easier to attach videos and pictures to illustrate a point.
4	Safer in the prevailing COVID -19 pandemic	It is safer especially in this prevailing COVID-19 pandemic.<br>A novel method.

.

Table 6. Shows the themes and subthemes for those who indicated that they did not enjoy online teaching (51.7%, n=15) in response to the question whether one enjoyed teaching online. They enumerated some of the disadvantages of online teaching such as clinical teaching and difficulty to give timeous feedback to students.

Eighty-three per cent (83%, n=24) of faculty had commenced online teaching and learning with 17% (n=5) yet to commence teaching online.

Table 6. Shows themes and subthemes in response to the question whether one enjoyed teaching online.

Number	Theme	Sub-theme
1	Lack of physical interaction with students	I am comfortable with my students<br>It lacks interaction<br>You cannot see students as you teach so you don't know if what you deliver is effective and understood<br>I prefer physical interaction with students<br>Less interaction with students<br>I teach better with face –to- face teaching
		I do not link with 60% of my students as they depend on other students as they re-route information via other platforms like WhatsApp<br>I cannot see students as they switch off their videos
2	Frequent disruption of lecture due to network challenges	Internet connectivity not good<br>This is a whole new experience which is marred by lack of suitable internet connectivity, lack of data and resources.<br>Network challenges can be a problem<br>I often have internet problems leading to disruption in teaching

3	Lack of immediate feedback	Student facial response and engagement allows me to continuously modify my teaching<br>It is not possible to assess facial expressions to see if students understood.<br>There is lack of immediate feedback.
4	Unsuitable for practical based learning	Online learning is unsuited for practical based learning

Table 7, shows the platforms in use for online teaching. The most commonly used platform is ZOOM (62.1%, n=18).

Table 7. Platforms used for online teaching

Platform	N	%
Zoom	18	6..2
Moodle	5	17.2
E-Mhare	2	6.9
Google meet	1	3.4
Google classes	1	3.4
Zoom and Google	1	3.4
WhatsApp and emails	1	3.4
Totals	29	100

Table 8. Shows barriers to online teaching, learning and assessment and possible solutions**.** Internet connectivity, high data costs, and inability to pass on technical skills were some of the barriers to online teaching. Solutions offered included a subsidy to faculty to access data and incorporate facilities that would beam online activities in the wards.

Table 8. Shows the barriers and possible solutions to online teaching and learning

Barrier	Possible Solutions
1.University internet connectivity may be unavailable, disrupted or broken down	Lecturers and students should be supported financially so that they secure data for efficient uninterrupted flow of lectures.
2. Non-availability of allowances for internet for lecturer	Provide data or allowance for internet to lecturers to enable smooth lecture planning and teaching off campus.
3. Unable to pass on technical skills as bedside teaching is impossible	To accommodate physical lectures in wards in turn with online teaching.
4. Data costs too high if venue of teaching is not the workplace.	To provide lecturers with internet allowance.
5. Lectures disrupt by use of the limited 40 minute zoom session and time wasted when re-logging in.	The institution to subscribe to the unlimited facility.
6.Unvavailability of internet equipment in the units	The purchase of internet equipment by the institution

Inattention by students	More training is required about the monitoring of student attendance and participation.

DISCUSSIONS

There is a general agreement that online learning is a good option for acquiring theoretical knowledge since it is not possible to hold face-to-face learning under the COVID-19 pandemic. Efficacy of online learning has long been acknowledged [29 -34]. Hugenholtzel found that e-learning is just as effective in enhancing knowledge as lecture-based learning [31]. A study indicated that online learning was less effective in building skills and knowledge mainly due to the difficulty of interacting with peers and faculty along with the acquisition of skills which are mostly better acquired through in-person interaction [7]. However, literature indicates that high level of satisfaction are related to well -structured and organised e-courses which also have a greater impact on knowledge accumulation and student performance compared with traditional learning [7]. Systematic reviews have reported that blended learning has better effects on knowledge outcomes compared to traditional learning [33, 36].

Blended learning has been adopted by our institution with small groups of students attending clinical settings and in-person lectures intermixed with online learning. In the current study the majority of faculty (65.5%) had received training in online teaching, learning and assessment. In line with other studies in the literature the technological skills to provide online courses increases the educational value of faculty. [37]. There is need to continue to support faculty as they implement e-learning.

Twenty-nine (29) faculty out of 100 who were invited participated in the study 62.1% (n=18) were males and 37.9% were females. The majority of the respondents were in the lecturer (37.9%) and senior lecturer grade (34.5%). This could reflect a relatively low experience in teaching and possibly age hence their perception of e-learning is likely to be positive based on their high technology capacity. This could also explain their enjoyment of online teaching, learning and assessment. Fischer et al. stated that older staff with long traditional teaching experience usually have limited interaction with technology and lacking the development of their necessary skills [38,39].

The most commonly used device for the delivery of teaching was the laptop (82.7%) followed by the personal computer (10.3%). The institution would then be expected to assist faculty with replacement of laptops as support for the continued online teaching, learning and assessment. Faculty most commonly used W-LAN (51.7%) followed by the LAN (34.5%).The institution would be expected to improve the internet connectivity at the institution and also assist faculty with data when they are away from the office so that they could continue with online teaching wherever they would be especially with the lockdown measures.

In agreement with other studies in the literature the majority of respondents (93.1%) believed that there were benefits in online teaching [37, 38]. Literature indicates that some of the factors among faculty that influence the level of satisfaction of online teaching are self-gratification, intellectual challenge, interest in using technology, and associated professional development opportunities [5,40]. This study found that the benefits of e-learning were: convenience for teaching, cost effectiveness, effective teaching approach and a safer teaching approach under the COVID-19 pandemic. Most institutions were using blended learning before the advent of the COVID-19 pandemic and did find the switch to completely online overwhelming.

Six-point nine percent (6.9%) did not agree that there were any additional benefits compared with face-to-face teaching and that immediate feedback is curtailed, lack of physical interaction with students, network challenges affecting lecture delivery and suitability for practical-based learning, and negative impact on hands on training or skills transfer. They also implored for the need to hold regular training courses on online teaching and learning. This is in agreement with other studies in the literature that showed that some faculty were not fully comfortable with e-learning as a teaching tool and attributed this to factors such as technological challenges, difficulty in interactions and discussions with students, lack of adequate internet connectivity, students access to internet was difficult and expensive, and personal learning preference [37,38].There is need to cultivate a culture of change as institutions transition to online teaching, learning and assessment as this will reduce resistance. Advanced planning and infrastructure are important for successful implementation of e-learning [41]. There is also need to modulate the traditional methods of teaching, learning and assessment for e-learning.

The majority of faculty did not agree that online teaching enhanced their teaching capacity while those who believed in the enhancement from online teaching felt that it added value to the learning of the students. They indicated the need for further training and adequate appropriate tools and reliable internet connectivity.

The majority (83%; n=24) are already participating in online teaching with zoom being the most common platform used (62.1%) with social media and email being used to communicate with students. Other platforms

were also used: Google meet, Google lessons, e-Mhare. In our faculty both synchronous (live or in real-time) and asynchronous (recorded or self-paced) are used through the University's learning management system (along with Zoom or Microsoft Teams). Synchronous e-learning is offered in the form of interactive teaching and clinical case discussions in small and large group formats [37] and asynchronous e-learning includes preparation of course materials for students in advances to be accessed later (recorded lectures, supportive videos, external links for recommended websites, and electronic books [37,41].

Literature indicates that there are many significant barriers to the adoption and implementation of e-learning by medical schools [5, 10]. These barriers include technology/infrastructure barriers, institutional/educators' and student barriers [19]. In agreement with other studies from LMIC lack of infrastructure, advanced technology, internet access, and poor quality of internet services (internet band width and connectivity limitations), limited technical support, unfamiliarity with e-learning systems were mentioned as barriers to the transition to virtual teaching/training and learning [15,19,20] : unavailability of university internet connectivity (unavailable, disrupted, broken down, interrupted power supply), absence of financial allowances to pay for internet service data (high cost) , inattention by students, inability to perform skills transfer , disturbances from the home environment (working at home due to lockdown measures). The disturbances at home are similar to those in other studies which showed that the learning environment at home posed the greatest challenge while technological literacy and competency was the least of their challenges [34].Low household income in LIMC makes it difficult for one to access the necessary equipment for online learning, along with poor power supply, poor internet connectivity, sociocultural dynamics as gender roles, educators' and learners' competence related to online teaching.

Blended teaching and learning, combining online and face-to-face teaching, balances benefits and drawbacks of online and face-to-face teaching [30]. Distance online course can prepare students as well as face-to-face. However, performance in online learning is reduced due to the COVID-19 driven social distancing and physical isolation as activities such as workshops, laboratories, and clinical rotations have had to be suspended [30].

The teaching environment poses challenges met by faculty inclusive of issues such as distractions at home (e.g., visitors, noise, and home chores) and limited space and facilities [34]. Poor internet connectivity, poor picture quality of videos and poor performing platforms were noted as possible barriers [25].Students and faculty need continuing training on online teaching and learning. A needs assessment should be performed among the students, health professions educators and the institution so that the identified needs and quality control be implemented at commencement of online teaching [35, 36, 42]. Poor internet connectivity, poor picture quality of videos and poor performing platforms were noted as barriers in this study as was noted in other studies [25].The needs assessment would help in the development of appropriate policies and a supportive culture.

Conclusions

The impact of the emergency embarking on online learning is not yet known since its early stages yet to assess the role of the academics on the future of e-learning in global health professions education. These findings can be used to improve health professions faculty approach to online teaching and learning as the global COVID-19 pandemic appears to be with us for a long time to come.

Institutions should improve the online teaching and learning infrastructures: learning management system (webinar facilities (zoom etc.) online learning platforms, mobile applications, Skype, Google Hangouts, training of both students and faculty on online teaching and learning, simulation/skills, laboratories, develop online curriculum, develop online examinations, telehealth, online video libraries. The developments should be embarked upon in line with their unique challenges of their faculty and the institutional existing online infrastructures. Continuing training and support on e-learning for faculty is important for successful acceptance and implementation of e-learning.

Methods of introducing and supporting health professions faculty transitioning to new teaching and learning circumstances and environments should provide contextualised both online and in-person strategies. Consideration of the negative and positive aspects of e-learning should allow for the development of a blended learning approach, flipped class, team-based learning approach that integrates aspects of face-to-face learning and e-learning to achieve the intended learning outcomes especially in practical and clinical training.

REFERENCES:

1. WHO (2020). WHO Director- General's Opening Remarks at the Media Briefing on COVID-19- 11 March 2020. Available online at: https://www.who.int/director-general/speeches/detail/who-director general-s-opening-remarks-at-the-media-briefinh-on-covid-19- 11-March-2020. (Accessed September 30:2021.

2. Watermeyer R, Crick T, Knight C, Goodall J. COVID-19 and digital disruption in UK universities: afflictions and affordances of emergency online migration. Higher Education.2021; 81:623-641.

3. The Lancet. Editorial 2020: Redefining vulnerability in the era of COVID-19. https://doi.org/10.1016/50140-6736 (20)30757-v

4. UNESCO COVID-19. Educational disruption and response 2020. (Available from https: //en. unesco.org /themes/education-emergencies/coronavirus-school-closure.

5. Rad FA, Otaki F, Baqain Z, et al. Rapid transition to distance learning due to COVID-19: Perceptions of postgraduate dental learners and instructors. PLOS ONE. 2021.https://doi.org/10.137/journal.pone.0246584.

6. Kernohan D. Which universities are moving to remote teaching? WONKHE 2020: https: // wonke.com/ blogs/which-universities-are-moving-to-remote-teaching/

7. Al Qhtani A, Al Swedan N, Almulhim A. et al. Online versus classroom teaching for medical students during COVID-19: measuring effectiveness and satisfaction. BMC Med Educ.2021; 21: 452-8.

8. Crawford J, Butler-Henderson K, Rudolph J, et al. M. COVID-19: 20 Countries' higher education intra-period digital pedagogy responses. J Appl. Learn. Teach2020; 3:1 340-389.https;//journal.sfu.ca/jalt /index. php /jalt / index1-

9. Adman M, Anwar K. Online learning amid the COVID-19 pandemic: students 'perspectives. Journal of Pedagogical Sociology and Psychology.2020; 2: 45-51.

10. Kaur G. Digital life: Boon or bane in teaching sector on COVID-19. CLIO an Annual Interdisciplinary Journal of History.2020; 6: 416-427.

11. Schlenz MA, Schmidt A, Wostmann B et al.Students' and lecturers' perspectives on the implementation of online learning in dental education due to SARS-CoV-2 (COVID-19): a cross-sectional study. BMC Medical Educ2020; 20:354-361.

12. Ebner M, Schon S, Braun C et al. COVID-19 pandemic as E-learning boost? Chronological development and effects at an Austrian University against the background of the concept of "E-learning Readiness". Future Internet2020; 12:354-371.

13. Tabatabai S. Simulations and virtual learning supporting clinical education during the COVID-19 pandemic. Adv Medic Educ and Pract2020; 11:513-516.

14. Ahmed H, Alf M, Elghazaly H. COVID-19 and medical education. Lancet.2020; 20:777-778.

15. Kiguli-Malwadde E. COVID-19 and its impact on health professions education in Africa. Afr Health.2020; 13:12-13.

16. Papapanou M, Routsi E, Tsamkis K et al. Medical education challenges and innovations during COVID-19 pandemic. Postgrad Med J.doi:1136/postgrad 2021-140032.

17. Doherty J.Education and training update: e-learning in clinical teaching. Brit J Hosp Med. 2010; 71:44-47.

18. Wang Y, Rongbin Y, Liu Y et al.Qian W (2021). Students' and Teachers' perspective on the implementation of medical education in China: a qualitative study. Adv Med Educ Pract 12; 895-902.

19. Al-Balas M, Al-Balas H I, Jaber HM et.al. Distance learning in clinical medical education amid COVID-19 pandemic in Jordan: current situation, challenges, and perspectives 2020; 20: 341-8.

20. Gismalla MDA, Mohamed MS, Ibrahim OSO et. Al. Medical students' perception towards e- learning during COVID-19 pandemic in a high burden developing country 2021; 21:3 77-13.

21. Olum R, Alulinda L, Kigozi E et.al. Medical education and e-learning during the COVID-19 pandemic: awareness,

attitudes, preferences, and barriers among undergraduate medicine and nursing students at Makerere University, Uganda.2020; 7:1-9.

22. Li W, Gillies R, He M et al. Barriers and facilitators to online medical and nursing education during the COVID-19 pandemic: perspectives from international students from low- and middle- income countries and their teaching staff. Hum Resourc Health 2021;19:64-78.https://doi.org/10.1186/3129960-021-00609-9

23. Panahl P, Borna F.Distance learning challenges: challenges, new solution 2014 37th International Convention on Information and Communication Technology .Opatija: Electronics and Microelectronics (MIPRO); 2014. P653-6. https://doi.org/10.1109/MIPRO.20146859648.20.

24. Dawidziuk A, Kawkka M, Szyszka B et al. Global access to technology-enhanced medical education during the COVID-19 pandemic: the role of students in narrowing the gap. 2021; 9:S1-S5.

25. Di Giano P, Di Paolo C. COVID-19 and dental distance-based education: students' perception in an Italian University. BMC Med Educ.2021; 21:414-9.

26. Sigdel S, Ozaki A, Dhakal R, et al. Medical education in Nepal: Impact and challenges of the COVID-19 pandemic. Acad Med 2021; 96: 340-342.

27. Xin LJ, Hathim AAH, Yi NJ et al. Digital learning in medical education: comparing experiences of Malaysian and Japanese students. BMC Med Educ 2021; 21: 418-9.

28. Kiesewetter J, Sailer M, Jung VM et al. learning clinical reasoning how virtual patient case format and prior knowledge interact. BMC Medl Educ.2020;20;73. https://doi.org/10.1186/s12909-020-1987-y.

29. Dost S, Hossain A, Shehab M et al. Perceptions of medical students towards online teaching during the COVID-19 pandemic: a national cross-sectional survey of 2721 UK medical students. BMJ Open 2020;10: e042378.doi:10.1136/bmj/open-2020-042378.

30. Rossettini G, Geri T, Turolla A et al. Online teaching in physiotherapy education during COVID-19 pandemic in Italy: a retrospective case-control study on students' satisfaction and performance. BMC Med Educ 21;2021; 21:456-12.

31.Hugenholtz NI, de Groun EM,Smits PB et al. Effectiveness of e-learning in continuing Vallee A, BlacherJ,Carlou A. Sorbets E. Blended learning compared to traditional learning in medical education: systematic review and meta-analysis. J Med Internet Res.2020; 22(8)e16504.http: //doi.org/10.2196/16504. PMID:32773378; PMCID:PMC7445617.

32. Wilcha RJ. Effectiveness of virtual medical teaching during the COVID-19 crisis. Systematic Review.JMIR MedEdu.2020; 6(2): e20963.https://doi.org/10.21963.PMID:33106227:PMCID:PMC7682786.

33. Baherimoghadam T, Hamedani S, Mehrabi M et al. The effect of learning style and general self-efficacy on satisfaction of e-learning in dental students. BMC Med Educ 2021; 21: 463-9.

34. Barrot JS, Linares II, Del Rosario LS. Students' online learning challenges during the pandemic and how they cope with them: the case of the Phillippines. Educ Infor Tech. https://doi.org/10.1007/s10639-021-10589-x.

35. Ardekani A, Hosseini SA, Tabari P et al. Student support systems for undergraduate medical students during the COVID-19 pandemic: a systematic narrative review of the literature. BMC Med Edu. 2021; 21:352-64.

36. Ashoka B, Ong SY, Tay KH et al. Coordinated responses of academic medical centres to pandemics: sustaining medical education during the COVID-19 pandemic. Med Teacher. https://doi.or /10.1080/01422159X. 2020. 1757634.

37. Zalat MM, Hamed MS, Bolbot SA. The experiences, challenges, and acceptance of e-learning as a tool for teaching during the COVID-19 pandemic among university medical staff. 2021. PLoS ONE 16(3): e 0248758. https://doi.org/10.1371/journal.pone0248758.

38. Hong KS, Lai KW, Holton D (2004). Students. Satisfaction and perceived learning with a web- based course. J Educ Tech Soc 2004; 6:1-12.

39. Fischer SH, David D, Crotty BH et al. (2014). Acceptance and use of health information technology by community-dwelling elders. Int J Med Infor 2014; 83: 624-35.

40. Bolliger DU, Wasilik O. Factors influencing faculty satisfaction with online teaching and learning in higher education. Dist Educ 2009; 30: 103-16.

41. Almuwals A, Alqabbani S, Benajiba N et al. (2021). An emergency shift to e-learning in health professions education: a comparative study of perspectives between students and institution. Int J Learn Teach Educ Res2021; 20: 16-37.

42. Mishra L, Gupta T, Shree A. Online teaching-learning in higher education during lockdown period of COVID-19 pandemic 2020.Int Journal Edu Res Open. https://doi/10.1016/j.ljedro.2020.10012.

Revised Manuscript with Track Changes

PLOS ONE

Health professions faculty's perceptions of online teaching and learning during the COVID-19 pandemic

--Manuscript Draft--

Manuscript Number:	PONE-D-21-33695
Article Type:	Research Article
Full Title:	Health professions faculty's perceptions of online teaching and learning during the COVID-19 pandemic
Short Title:	Health professions educators' perceptions online teaching under COVID-19 pandemic
Corresponding Author:	Midion Mapfumo Chidzonga, BDS FFDRCSI<br>Professor<br>Harare, ZIMBABWE
Keywords:	COVID-19 pandemic; higher education; online teaching, learning and assessment, health professions faculty
Abstract:	The global societal impact of the COVID-19 pandemic is incalculable with profound social suffering, deep economic hardships and enforced closure of schools, businesses, and higher learning institutions through the imposition of lockdown and social distancing in mitigation of the spread of the SARS-Cov-2 infection. Institutions have had to hastily migrate teaching, learning and assessment to online domains, at times with ill-prepared academics, students and institutions and with unwelcome and disorienting consequences. Our study surveyed perspectives of faculty at the University of Zimbabwe Faculty of Medicine and Health Sciences (UZFMHS) towards the hastily adopted online teaching, learning and assessment implemented in response to the mitigation of the COVID-19 pandemic. Twenty-nine (29) faculty from the major disciplines and career hierarchy participated in the study ~~Twenty nine (29) faculty in all the major disciplines and career hierarchy.~~ There were mixed responses regarding the use of this modality for teaching, learning and assessment: training before online teaching, learning and assessment, advantages and disadvantages, cost effectiveness, effectiveness for teaching, learning and assessment, effect on student feedback, disruptions from internet connectivity issues, interaction with students, suitability for practical training, and barriers to online teaching, learning and assessment. These results would enable the UZFMHS develop institutional and personalised approaches that would enable execution of online teaching, learning and assessment under the current and post COVID-19 pandemic.
Order of Authors:	Midion Mapfumo Chidzonga, BDS FFDRCSI
	Clara Opha Haruzivishe, PhD
	Judith Rukweza, PhD
	Vasco Chikasha, PhD
Additional Information:	
Question	Response

Powered by Editorial Manager® and ProduXion Manager® from Aries Systems Corporation

**Financial Disclosure**<br><br>Enter a financial disclosure statement that describes the sources of funding for the work included in this submission. Review the submission guidelines for detailed requirements. View published research articles from *PLOS ONE* for specific examples.<br><br>This statement is required for submission and **will appear in the published article** if	1.All authors were under the same funding: initials are MMC, COH, JR, VC<br>2.www.norad.no<br>3.Funded by the Norwegian Programme for Capacity Development in Higher Education and Research Development<br>(NORHED)<br>4.NO.The funders had no role in study design, data collection and analysis, decision to publish, or preparation of the manuscript
the submission is accepted. Please make sure it is accurate.<br><br>**Unfunded studies**<br>Enter: *The author(s) received no specific funding for this work.*<br><br>**Funded studies**<br>Enter a statement with the following details:<br>• Initials of the authors who received each award<br>• Grant numbers awarded to each author<br>• The full name of each funder<br>• URL of each funder website<br>• Did the sponsors or funders play any role in the study design, data collection and analysis, decision to publish, or preparation of the manuscript?<br>• **NO** - Include this sentence at the end of your statement: *The funders had no role in study design, data collection and analysis, decision to publish, or preparation of the manuscript.*<br>• **YES** - Specify the role(s) played.<br><br>* typeset	

Powered by Editorial Manager® and ProduXion Manager® from Aries Systems Corporation

**Competing Interests**<br><br>Use the instructions below to enter a competing interest statement for this submission. On behalf of all authors, disclose any competing interests that could be perceived to bias this work—acknowledging all financial support and any other relevant financial or non-financial competing interests.<br><br>This statement is required for submission and **will appear in the published article** if the submission is accepted. Please make sure it is accurate and that any funding sources listed in your Funding Information later in the submission form are also declared in your Financial Disclosure statement.<br><br>View published research articles from *PLOS ONE* for specific examples.	NO. The authors have declared that no competing interests exist.

**NO authors have competing interests**<br><br>Enter: *The authors have declared that no competing interests exist.*<br><br>**Authors with competing interests**<br><br>Enter competing interest details beginning with this statement:<br><br>*I have read the journal's policy and the authors of this manuscript have the following competing interests: [insert competing interests here]*<br><br>* typeset	

Powered by Editorial Manager® and ProduXion Manager® from Aries Systems Corporation

**Ethics Statement**<br><br>Enter an ethics statement for this submission. This statement is required if the study involved:<br><br>• Human participants<br>• Human specimens or tissue<br>• Vertebrate animals or cephalopods<br>• Vertebrate embryos or tissues<br>• Field research<br><br>Write "N/A" if the submission does not require an ethics statement.<br><br>General guidance is provided below. Consult the submission guidelines for detailed instructions. **Make sure that all information entered here is included in the Methods section of the manuscript.**	Conflicts of interest/Competing interest: None<br>Availability of data and material (data transparency): Available<br>Code availability (software application or custom code): Not applicable<br>Authors' contributions (optional)<br>Ethics approval: Permission to carry out the study at the University of Zimbabwe Faculty of Medicine and Health Sciences (UZFMHS) was granted by the Dean of the UZFMHS. Ethical clearance to carry out the study was obtained from the local ethical review board, Joint Research Ethics Committee of the UZFMHS and Parirenyatwa Group of Hospital (JREC) and the Medical Research Council of Zimbabwe (MRCZ).Informed consent was obtained from the participants before participation in the study. No risks to the participants were envisaged during the study process. Benefits to the participants may accrue through their perspectives being used to enrich the implementation of online teaching, learning and assessment.<br>Participation was voluntary and participants were requested to read the consent form along with the information sheet and only proceed with filling in the form if they were agreeable with the information given on the consent form and the information sheet.<br>Consent to participate: Participation was voluntary and participants were requested to read the consent form along with the information sheet and only proceed with filling in the form if they were agreeable with the information given on the consent form and the information sheet.<br>Consent for publication: Not applicable<br>Text formatting: 10-point Times Roman<br><br>Acknowledgements: Many thanks to the NORHED for funding this project.

Powered by Editorial Manager® and ProduXion Manager® from Aries Systems Corporation

**Format for specific study types**<br><br>**Human Subject Research (involving human participants and/or tissue)**<br>• Give the name of the institutional review board or ethics committee that approved the study<br>• Include the approval number and/or a statement indicating approval of this research<br>• Indicate the form of consent obtained (written/oral) or the reason that consent was not obtained (e.g. the data were analyzed anonymously)<br><br>**Animal Research (involving vertebrate animals, embryos or tissues)**<br>• Provide the name of the Institutional Animal Care and Use Committee (IACUC) or other relevant ethics board that reviewed the study protocol, and indicate whether they approved this research or granted a formal waiver of ethical approval<br>• Include an approval number if one was obtained<br>• If the study involved *non-human primates*, add *additional details* about animal welfare and steps taken to ameliorate suffering<br>• If anesthesia, euthanasia, or any kind of animal sacrifice is part of the study, include briefly which substances and/or methods were applied<br><br>**Field Research**<br><br>Include the following details if this study involves the collection of plant, animal, or other materials from a natural setting:<br>• Field permit number<br>• Name of the institution or relevant body that granted permission	
**Data Availability**<br><br>Authors are required to make all data underlying the findings described fully available, without restriction, and from the time of publication. PLOS allows rare exceptions to address legal and ethical concerns. See the PLOS Data Policy and FAQ for detailed information.	Yes - all data are fully available without restriction

A Data Availability Statement describing where the data can be found is required at submission. Your answers to this question constitute the Data Availability Statement and **will be published in the article**, if accepted.<br><br>**Important:** Stating 'data available on request from the author' is not sufficient. If your data are only available upon request, select 'No' for the first question and explain your exceptional situation in the text box.<br><br>Do the authors confirm that all data underlying the findings described in their manuscript are fully available without restriction?	
**Describe where the data may be found in full sentences. If you are copying our sample text, replace any instances of XXX with the appropriate details.**<br><br>• If the data are **held or will be held in a public repository**, include URLs, accession numbers or DOIs. If this information will only be available after acceptance, indicate this by ticking the box below. For example: *All XXX files are available from the XXX database (accession number(s) XXX, XXX.*).<br>• If the data are all contained **within the manuscript and/or Supporting Information files**, enter the following: *All relevant data are within the manuscript and its Supporting Information files.*<br>• If neither of these applies but you are able to provide **details of access elsewhere**, with or without limitations, please do so. For example:<br><br>*Data cannot be shared publicly because of [XXX]. Data are available from the XXX Institutional Data Access / Ethics Committee (contact via XXX) for researchers who meet the criteria for access to confidential data.*<br><br>*The data underlying the results presented in the study are available from (include the name of the third party*	Not applicable

Powered by Editorial Manager® and ProduXion Manager® from Aries Systems Corporation

and contact information or URL).<br>• This text is appropriate if the data are owned by a third party and authors do not have permission to share the data.<br><br>* typeset	
Additional data availability information:	Tick here if your circumstances are not covered by the questions above and you need the journal's help to make your data available.

Powered by Editorial Manager® and ProduXion Manager® from Aries Systems Corporation

Click here to access/download;Manuscript;Health Professions COVID-19.docx

TITLE PAGE

Title: Health professions faculty's perceptions of online teaching and learning during the COVID-19 pandemic

1. Midion Mapfumo CHIDZONGA

Department of Oral Health

Faculty of Medicine and Health Sciences

University of Zimbabwe

HARARE

ZIMBABWE

mtmchidzonga@yahoo.com

ORCID http://orcid.org/0000-0002-4574-1158

2. Clara HARUZIVISHE

Department of Primary Health Care

Unit of Nursing Sciences

Faculty of Medicine and Health Sciences

University of Zimbabwe

HARARE

ZIMBABWE

claraopha@gmail.com

3. Vasco CHIKWASHA

Unit of Global and Public Health

Department of Primary Health Care

Faculty of Medicine and Health Sciences

University of Zimbabwe

HARARE

ZIMBABWE

vchikasha@gmail.com

4. Judith RUKWEZA

Nursing Sciences

Department Primary Health Care

Faculty of Medicine and Health Sciences

University of Zimbabwe

HARARE

ZIMBABWE

rukwezajudith@gmail.com

Corresponding Author: Midion Mapfumo Chidzonga

mtmchidzonga@yahoo.com

ABSTRACT

The global societal impact of the COVID-19 pandemic is incalculable with profound social suffering, deep economic hardships and enforced closure of schools, businesses, and higher learning institutions through the imposition of lockdown and social distancing in mitigation of the spread of the SARS-Cov-2 infection. Institutions have had to hastily migrate teaching, learning and assessment to online domains, at times with ill-prepared academics, students and institutions and with unwelcome and disorienting consequences. Our study surveyed perspectives of faculty at the University of Zimbabwe Faculty of Medicine and Health Sciences (UZFMHS) towards the hastily adopted online teaching, learning and assessment implemented in response to the mitigation of the COVID-19 pandemic. Twenty-nine (29) faculty from the major disciplines and career hierarchy participated in the studyTwenty nine (29) faculty in all the major disciplines and career hierarchy. There were mixed responses regarding the use of this modality for teaching, learning and assessment: training before online teaching, learning and assessment, advantages and disadvantages ,costdisadvantages, cost effectiveness, effectiveness for teaching, learning and assessment, effect on student feedback, disruptions from internet connectivity issues, interaction with students, suitability for practical training, and barriers to online teaching, learning and assessment. These results would enable the UZFMHS develop institutional and personalised approaches that would enable execution of online teaching, learning and assessment under the current and post COVID-19 pandemic.

Keywords: COVID-19 pandemic; higher education; online teaching, learning and assessment, health professions faculty.

DECLARATIONS

Funding: Norwegian Programme for Capacity Development in Higher Education and Research Development (NORHED)

Conflicts of interest/Competing interest: Not applicable

Availability of data and material (data transparency): Available

Code availability (software application or custom code): Not applicable

Authors' contributions (optional)

Ethics approval: Permission to carry out the study at the University of Zimbabwe Faculty of Medicine and Health Sciences (UZFMHS) was granted by the Dean of the UZFMHS. Ethical clearance to carry out the study was obtained from the local ethical review board, Joint Research Ethics Committee of the UZFMHS and Parirenyatwa Group of Hospital (JREC) and the Medical Research Council of Zimbabwe (MRCZ). Informed consent was obtained from the participants before participation in the study. No risks to the participants were envisaged during the study process. Benefits to the participants may accrue through their perspectives being used to enrich the implementation of online teaching, learning and assessment.

Participation was voluntary and participants were requested to read the consent form along with the information sheet and only proceed with filling in the form if they were agreeable with the information given on the consent form and the information sheet.

Consent to participate: Participation was voluntary and participants were requested to read the consent form along with the information sheet and only proceed with filling in the form if they were agreeable with the information given on the consent form and the information sheet.

Consent for publication: Not applicable

Text formatting: 10-point Times Roman

Automatic page numbering

Acknowledgements: Many thanks to the NORHED for funding this project.

INTRODUCTION: The World Health Organisation (WHO) declared COVID-19 a global public health emergency of international concern on 30th January 2020 and subsequently declared it a pandemic on 11th of March [1, 2]. The societal impact of COVID-19 is incalculable as the pandemic continues to cause profound social suffering and deep economic hardships especially for society's most vulnerable and less fortunate [1,2,3]. Many aspects of life have been seriously disrupted by the COVID-19 pandemic. The lockdown measures, in a bid to mitigate the spread of the SARS-Cov-2 infection, have led to enforced closure of schools, colleges, and universities, but without cessation of all learning, teaching and assessment although many forms of assessment have had to be suspended [2,4]. Most institutions of higher learning had to hastily migrate to the online domains with some of them with poor online infrastructures as well as ill-prepared academics and students [5, 6, and 7]. The new experience has been ~~unwelcome~~unwelcoming and disorienting to some educators, and students alike. The pandemic has also exposed deficiencies in the use of technology in higher education, which has long been debated, and is now overdue [2~~].The~~]. The University of Zimbabwe Faculty of Medicine and Health Sciences (UZFMHS) now has to embrace this change hastily. This new challenge along with the attendant obstacles has to be faced by many higher education institutions [8].There is no indication when the pandemic will be contained and as such educational institutions across the globe have now opted to use the available technical resources to create online teaching, learning and assessment material for students of all academic levels [9,10].The pandemic has thus created a forced digitalisation change, which was long overdue, and an opportunity to accelerate digital transformation, in health professions education [11,12].This is a positive move post COVID-19 pandemic. Pre- clinical health professions education can be taught through webinars. Unfortunately, online teaching, learning, and assessment is most useful for theoretical content but innovative ways have had to be developed to enable clinical training and assessment [13,14,15,16].It is known that formative and high-stakes examinations could be carried out by modifying the current assessment processes such as multiple choice questions, short-answer questions can be done online at the same time viva voce, teleconferencing between candidate and examiners; use of simulation/ objective structured clinical examination; modify patient contact stations to simulated patients; task-trainers and hybrid simulations; patient requiring components; use of personal protective equipment (PPE) at each station; examiner view the examination process remotely .Clinical training depends heavily on hands-on- patient contact to enable clinical skills acquisition, practical work in the laboratories which would require advanced technology which is currently unavailable in most low and middle income countries (LMICs). However, collaboration with high income countries (HIC) may alleviate this situation. Innovative approaches are being put in place to enable continued clinical training: vaccination of all students and faculty; provision of adequate PPE to ~~students ,faculty~~students, faculty and patients; good hand hygiene; mask wearing; face shields; social distancing; full-body PPE(long-sleeved disposable gown, eye protection, gloves) for aerosol producing procedures; and small numbers of students at a time. For non-aerosol producing procedures, minimum PPE is provided (disposable apron, facemask, gloves and eye protection is provided. This ensures that the safety of patients, students and staff is not compromised.

Health professions students need to be exposed to patients in an authentic environment, an apprenticeship type of learning. This is usually in hospital wards, outpatient clinics and simulated teaching laboratories. Under the current COVID-19 pandemic live broadcasts, films or virtual case presentations, and surgical procedures might be used to complement the limited access to patients.

Faculty are expected to increase their digital competence so as to come up with innovative virtual teaching, learning, and assessment tools.

The COVID-19 pandemic has curtailed clinical placements as well as elective services offered in clinical services. However, there is need to continue to adequately train health professionals to run the healthcare system. Student clinical placement has to continue while observing safe supervision of students, adequate provision of PPE, social distancing, washing and sanitising hands. Various learning management systems are available: Moodle, Massive Open Online Courses. Several tools are also available that allow for video communications, video and audio conferencing, chats, and webinars: Skype, Zoom, and Google Hangouts Meet, Google school [13].It is possible to set up real-time classroom experience that simulates patient encounters using live HD-quality mobile or fixed-cameras that ensures that all distance learners can collaborate at an equivalent baseline [14].Current virtual learning management systems offer access to educational content from anywhere, synchronously or asynchronously with interactive simulation learning [13].Unfortunately the theoretical knowledge has the challenge of not being able to be transferred to patient management. The way forward would be to introduce interactive simulation learning (computerised simulation education) which unfortunately need massive infrastructure ranging from clinical simulation management software and hardware, design and planning tools, file backup, cloud-based eLearning, and expert teams to provide support to counsellor education, case developers,

and virtual patient training [13]. This study at the UZFMHS surveyed the facu[illegible] teaching, learning and assessment. The information gathered would enable de[illegible] tailor made based the perceptions of faculty.

clara

clara
2022-05-04 03:25:18

The tool used to collect data was piloted on 5 faculty who were excluded from the main study. Following this pilot some items were removed and only relevant items were left: three 3 questions relating to demographic data and 13 questions relating to online teaching and learning were finally used for [illegible]

METHODOLOGY

SETTING: The study was conducted at the UZFMHS which provides health p[illegible] to various healthcare cadres.

STUDY DESIGN: Mixed method design.

ETHICAL CONSIDERATIONS: Permission to carry out the study was [illegible] UZFMHS. Ethical clearance was obtained from the local ethical review board, Joint Research Ethics Committee of the UZFMHS and Parirenyatwa Group of Hospital (JREC) and the Medical Research Council of Zimbabwe (MRCZ).Informed consent was obtained from the participants. Participation was voluntary. There were no risks to the participants. Participation was voluntary and participants were requested to read the consent form and only proceed with filling in the form if they were agreeable with the information given on the consent form. Benefits to the participants may accrue should their perspectives be used to enrich the development of online teaching, learning and assessment.

STUDY PARTICIPANTS: The participants were faculty of medicine, dentistry, nursing, physiotherapy, occupational therapy, pharmacy, health promotion and education, radiographers and medical laboratory scientists.

SAMPLING: [illegible]plines was selected from those who were accessible [illegible]ampus during the time of data collection. Only 29 faculty responded.~~We invited faculty in the above disciplines to participate.~~

STUDY INSTRUMENTS: [illegible]s and emailed to faculty. The questionnaire captured demo[illegible]hics, academic grade, number of years in teaching, computer literacy, device used when delivering teaching type of internet connections used, whether one received training on online teaching and learning, benefits of online learning to students, role of online learning in enhancing teaching, belief in online teaching, cost effectiveness, convenience for teaching, disadvantages and advantages of online education, barriers to online teaching, and online teaching practice, and platforms used in teaching. The tool used to collect data was piloted on 5 faculty who were excluded from the main study. Following this pilot some items were removed and only relevant items were left: three 3 questions relating to demographic data and 13 questions relating to online teaching and learning were finally used for the study.

DATA ANALYSIS: Data collected through the online survey was exported to STATA 15.1 statistical software for cleaning, analysis, calculation frequencies and percentages for categorical data. Qualitative data is presented as themes and ~~subthemes .Frequency~~subthemes. Frequency responses to "YES" and "NO" items are presented in the tables.

RESULTS:

Twenty-nine (29) faculty responded, 62.1% (n=18) males and 37.9% (n=11) females. The teaching experience of faculty ranged from 2 years to 44 years. A total of 19 (65.5%) had facilitated online teaching and 10 (34.5 %) had not.

~~Twenty nine (29) faculty responded, 62.1% (n=18) males and 37.9% (n=11) females. The teaching experience of faculty ranged from 2 years to 44 years. Sixty five point five per cent (65.5%, n=19) had received training on online teaching and 34.5% had not received any training.~~

On belief in online teaching, Table 1, shows responses to "Yes and "No" questions regarding online teaching, learning and assessment with 65.5% of the respondents indicating that they had received training on online teaching, learning and assessment indicative of a possible positive participation in online teaching. However, 51.7% did not enjoy teaching online with 52.0% believing that online teaching does not enhance their teaching capacity.

Table 1. Shows answers to questions regarding online teaching and learning

QUESTIONS	YES		NO	
	N	%	N	%
Have you received training on online teaching and learning?	19	65.5	10	34.5
Do you enjoy online teaching and learning?	14	48.3	15	51.7
Do you believe in benefits of online learning to students?	27	93.1	2	6.9
Do you believe that online learning enhances your teaching capacity	14	48.3	15	15.7

QUESTIONS	YES %/N	NO %/N
Have you received training on online teaching and learning?	65.5%/19	34.5%/10
Do you enjoy online teaching and learning?	48.3%/14	51.7%/15
Do you believe in benefits of online learning to students?	93%/27	7%/2
Do you believe that online learning enhances your teaching capacity	48%/14	52%/15

Table 1. Shows answers to questions regarding online teaching and learning

Platform	N/%
Zoom	18/62.1%
Moodle	5/17.2%
E-Mhare	2/6.9%
Google meet	1/3.4%
Google classes	1/3.4%
Zoom and Google	1/3.4%
WhatsApp and emails	1/3.4%
Totals	29/100%

Table 7. Platforms used for online teaching

Those who did not believe that there were benefits in online teaching supported their views by that there were no additional benefits when compared to physical contacts with students and that students were less engaged and feedback is curtailed. They also pointed out the need for proper training on online teaching, requisite tools and internet access for both students and faculty as well as negative impact on hands on courses or skills transfer.

Table 2.sShows that te academic grades of faculty. Teaching experience of faculty ranged from 2 to 44 years. All the faculty members were comfortable using a computer for teaching and learning. The majority of the faculty respondents are in the lecturer and senior lecturer grade. This is a positive situation as this group is likely to be in the age group that is technology savvy and should be able to navigate the education technology with ease.

Table 2. Shows academic grade of faculty.

Grade	N	%
Professor	8	(27.6
Associate Professor	0	(0.0
Senior Lecturer	10	(34.5
Lecturer	11	(37.9
TOTAL	29	100

Grade	N (%)
Professor	8 (27.6%
Associate Professor	0 (0.0%)
Senior Lecturer	10 (34.5%)
Lecturer	11 (37.9%)
TOTAL	29 (100%)

Table 2. Shows academic grade of faculty.

Table 3. sShows that tthe devices used in delivery of teaching. The majority of faculty, 852.7%, deliver their lectures using laptops which means they could deliver their teaching at any location where there is internet connectivity. One person did not specify what device they used.

Table3. Shows devices for delivering teaching.

Device	N	%
Smartphone	0	0
Tablet	1	3.6
Laptop	24	85.7
Personal Computer	3	10.3
Total	28	100

Device	N (%)
Smartphone	0 (0%)
Tablet	1 (3.5%)
Laptop	24 (82.7%)
Personal Computer	3 (10.3%)
Other (device not indicated)	1 (3.5%)
Total	29 (100.0%)

Table3. Shows devices for delivering teaching.

Table 4. Shows that most (51.7%) faculty received their internet connection through W-LAN (wireless connection, local area network), followed (34.5%) by the LAN (connection).This enables them to conduct their teaching wherever there is wireless internet connectivity.

Table 4. Shows type of internet connection used.

Internet connection used	N	%
Mobile network	I	3.5
W-LAN (wireless connection)	15	51.7
LAN (cable connection)	10	34.5
Both WLAN and LAN	3	10.3
Total	29	100

Internet connection used	N (%)
Mobile network	I (3.5%)
W-LAN (wireless connection)	15 (51.7%)
LAN (cable connection)	10 (34.5%)
Both WLAN and LAN	3 (10.3%)
Total	29 (100%)

Table 4. Shows type of internet connection used.

Table 5. Shows themes and subthemes for those who enjoyed online teaching (48.3%, n=14) in response to the question whether one enjoyed online teaching along with disadvantages and advantages of online teaching, convenient for teaching, cost effective, and safe teaching modality under the COVID-19 pandemic.

Table 5. Shows themes and subthemes for those who enjoyed online teaching in response to the question whether one enjoyed online teaching

Number	Theme	Subtheme
1	Convenient for teaching	It is a convenient method<br>It is flexible<br>Effective with big numbers<br>More practical because one doesn't have to travel to teach<br>I can teach from my office without having to go to different lecture rooms for different year groups<br>It is convenient as I can continue with my teaching even when I am not physically in the country<br>Teaching can be done at a convenient time<br>It is suited for a busy clinician
2	Cost effective	It is cheaper<br>It cuts on transport cost<br>A lecture can be recorded for future us<br>Challenges are welcome
3	More effective teaching methods explored and applied	Gives room to explore other ways of teaching like simulation and videos<br>Helps facilitator simplify teaching and produce quality lecture presentations.<br>It is easier to attach videos and pictures to illustrate a point.
4	. Safer in the prevailing COVID-19 pandemic	It is safer especially in this prevailing COVID-19 pandemic.<br>A novel method.

Table 5. Shows themes and subthemes for those who enjoyed online teaching in response to the question whether one enjoyed online teaching.

Table 6. Shows the themes and subthemes for those who indicated that they did not enjoy online teaching (51.7%, n=15) in response to the question whether one enjoyed teaching online. They enumerated some of the disadvantages of online teaching such as clinical teaching and difficulty to give timeous feedback to students.

Eighty threeEighty-three per cent (83%, n=24) of faculty had commenced online teaching and learning with 17% (n=5) yet to commence teaching online.

Table 6. Shows themes and subthemes for those who indicated that they did not enjoy online teaching in response to the question whether one enjoyed teaching online.

Number	Theme	Sub-theme
1	Lack of physical interaction with students	I am comfortable with my students<br>It lacks interaction<br>You cannot see students as you teach so you don't know if what you deliver is effective and understood<br>I prefer physical interaction with students<br>Less interaction with students<br>I teach better with face –to- face teaching<br>I do not link with 60% of my students as they depend on other students as they re-route information via other platforms like WhatsApp<br>I cannot see students as they switch off their videos
2	Frequent disruption of lecture due to network challenges	Internet connectivity not good<br>This is a whole new experience which is marred by lack of suitable internet connectivity, lack of data and resources.<br>Network challenges can be a problem<br>I often have internet problems leading to disruption in teaching
3	Lack of immediate feedback	Student facial response and engagement allows me to continuously modify my teaching<br>It is not possible to assess facial expressions to see if students understood.<br>There is lack of immediate feedback.
4	Unsuitable for practical based learning	Online learning is unsuited for practical based learning

Table 6. Shows themes and subthemes for those who indicated that they did not enjoy online teaching in response to the question whether one enjoyed teaching online.

Table 7, shows the platforms in use for online teaching. The most commonly used platform is ZOOM (62.1%, n=18).

Platform	N/%
Zoom	18/62.1%
Moodle	5/17.2%
E-Mhare	2/6.9%
Google meet	1/3.4%
Google classes	1/3.4%
Zoom and Google	1/3.4%
WhatsApp and emails	1/3.4%
Totals	29/100%

Table 7, shows the platforms in use for online teaching. The most commonly used platform is ZOOM (62.1%, n=18).

Table 7.Platforms used for online teaching

Platform	N	%
Zoom	18	6..2
Moodle	5	17.2
E-Mhare	2	6.9
Google meet	1	3.4
Google classes	1	3.4
Zoom and Google	1	3.4
WhatsApp and emails	1	3.4
Totals	29	100

Table 7.Platforms used for online teaching

Table 8. Shows barriers to online teaching, learning and assessment and possible solutions**.** Internet connectivity, high data costs, and inability to pass on technical skills were some of the barriers to online teaching. Solutions offered included a subsidy to faculty to access data and incorporate facilities that would beam online activities in the wards.

Table 8. Shows the barriers and possible solutions to online teaching and learning

Formatted: Indent: Left: 0"

Barrier	Possible Solutions
1.University internet connectivity may be unavailable, disrupted or broken down	Lecturers and students should be supported financially so that they secure data for efficient uninterrupted flow of lectures.
2. Non-availability of allowances for internet for lecturer	Provide data or allowance for internet to lecturers to enable smooth lecture planning and teaching off campus.
3. Unable to pass on technical skills as bedside teaching is impossible	To accommodate physical lectures in wards in turn with online teaching.
4. Data costs too high if venue of teaching is not the workplace.	To provide lecturers with internet allowance.
5. Lectures disrupt by use of the limited 40 minute zoom session and time wasted when re-logging in.	The institution to subscribe to the unlimited facility.
6.Unavailability of internet equipment in the units	The purchase of internet equipment by the institution
Inattention by students	More training is required about the monitoring of student attendance and participation.

~~2. Non-availability of allowances for internet for lecturer~~	~~Provide data or allowance for internet to lecturers to enable smooth lecture planning and teaching off campus.~~
~~3. Unable to pass on technical skills as bedside teaching is impossible~~	~~To accommodate physical lectures in wards in turn with online teaching.~~
~~4. Data costs too high if venue of teaching is not the workplace.~~	~~To provide lecturers with internet allowance.~~
~~5. Lectures disrupt by use of the limited 40 minute zoom session and time wasted when re-logging in.~~	~~The institution to subscribe to the unlimited facility.~~
~~6.Unvavailability of internet equipment in the units~~	~~The purchase of internet equipment by the institution~~
~~Inattention by students~~	~~More training is required about the monitoring of student attendance and participation.~~

~~Table 8. Shows the barriers and possible solutions to online teaching and learning~~

DISCUSSIONS

The lockdowns and subsequent closure of higher education institutions are mitigation measures to the spread of SARS-CoV-2 virus and protect the students, faculty and the community but at the same time disrupted the training of future healthcare professionals due to loss of time of learning/training and inability to continue adequate clinical training [15].The COVID-19 pandemic has caused the closure of university campuses around the world and migration of all learning, teaching, and assessment to online domains [2]. This has caused profound changes in health professions education with regards to clinical placements along with the social suffering and extensive economic hardships and strain on healthcare systems. High income countries (HIC) managed to swiftly migrate to e-learning with ease as they already had invested in digitalisation unlike the low and middle income countries (LMIC) that had minimal capacity and require extensive adjustments to achieve the abrupt switch to e-learning [1,17-22]. The e-learning technology has not been evenly dispersed throughout all nations and cultures [22].Limited institutional capacity and knowledge on online teaching inevitably made this transition more challenging for LMIC. The HIC successfully swiftly implemented a vast array of technology-enhanced learning solutions within the pre-existing structures: webinars conducted via zoom, Skype, Google Hangouts, WebX; online learning platforms; mobile applications; 3-dimensional anatomy models; and online question banks [19, 23, 24]. In some LMIC distance e-learning had not been adopted as a modality of teaching within health professions education [19]. The availability of essential infrastructures, well-trained educators, advanced technologies, efficient institutional strategies and student related barriers represent major challenges in integrating distance e-learning in health professions education in LMIC [23-26]. With the uncertainty of returning to normal life after the containment of the pandemic e-learning will remain the modality of teaching, learning and assessment in higher education.

29 faculty out of 100 who were invited.

clara

Digital learning is a process of integrating technology-mediated synchronous and asynchronous approaches including assessments, assignments, and tutoring enabling learning without any time and location restrictions 15, 27, 28]. Digital learning has a few components including digital teaching materials, digital tools, digital delivery, and autonomous learning. Health professions education has adopted digital learning with through virtual course, simulation software and teleconferencing and are struggling to come up with suitable virtual teaching and learning for the clinical component of their training that requires the traditional patient contact in the hospital/clinic set up.

This will be removed

There is is a in general agreement that online learning is a good option for acquiring theoretical knowledge since it is not possible to hold face-to-face learning under the COVID-19 pandemic. Efficacy of online learning has long been acknowledged [29 -34]. Hugenholtzel found that e-learning is just as effective in enhancing knowledge as lecture-based learning [31]. A study indicated that online learning was less effective in building skills and knowledge mainly due to the difficulty of interacting with peers and faculty along with the acquisition of skills which are mostly better acquired through in-person interaction [7]. However, literature indicates that high level of satisfaction are related to well -structured and organised e-courses which also have a greater impact on knowledge accumulation and student performance compared with traditional learning [7]. Systematic reviews

have reported that blended learning has better effects on knowledge outcomes compared to traditional learning [33, 36].

Blended learning has been adopted by our institution with small groups of students attending clinical settings and in-person lectures intermixed with online learning.

clara
2022-05-04 03:35:03

this section will be removed also.

In the current study the majority of faculty (65.5%) had received training in online teaching, learning and assessment. In line with other studies in the literature the technological skills to [illegible] the educational value of faculty. [37]. There is need to continue to support faculty as they implement e-learning.

Twenty nine (29) lecturers participated in the study. Twenty nine (29) faculty out of 100 invited participated in the study 62.1% (n=18) were males and 37.9% were females. The majority of the respondents were in the lecturer (37.9%) and senior lecturer grade (34.5%). This could reflect a relatively low experience in teaching and possibly age hence their perception of e-learning is likely to be positive based on their high technology capacity. This could also explain their enjoyment of online teaching, learning and assessment. Fischer et al. stated that older staff with long traditional teaching experience usually have limited interaction with technology and lacking the development of their necessary skills [38,39].

The most commonly used device for the delivery of teaching was the laptop (82.7%) followed by the personal computer (10.3%). The institution would then be expected to assist faculty with replacement of laptops as support for the continued online teaching, learning and assessment.

Formatted: Normal

Formatted: Indent: Left: 0"

Formatted: Indent: First line: 0"

Faculty most commonly used W-LAN (51.7%) followed by the LAN (34.5%).The institution would be expected to improve the internet connectivity at the institution and also assist faculty with data when they are away from the office so that they could continue with online teaching wherever they would be especially with the lockdown measures.

In agreement with other studies in the literature the majority of respondents (93.1%) believed that there were benefits in online teaching [37, 38]. Literature indicates that some of the factors among faculty that influence the level of satisfaction of online teaching are self-gratification, intellectual challenge, interest in using technology, and associated professional development opportunities [5,40]. This study found that the benefits of e-learning were: convenience for teaching, cost effectiveness, effective teaching approach and a safer teaching approach under the COVID-19 pandemic. Most institutions were using blended learning before the advent of the COVID-19 pandemic and did find the switch to completely online overwhelming.

Six point nine percent (6.9%) did not agree that there were any additional benefits compared with face-to-face teaching and that immediate feedback is curtailed, lack of physical interaction with students, network challenges affecting lecture delivery and suitability for practical-based learning, and negative impact on hands on training or skills transfer. They also implored for the need to hold regular training courses on online teaching and learning. This is in agreement with other studies in the literature that showed that some faculty were not fully comfortable with e-learning as a teaching tool and attributed this to factors such as technological challenges, difficulty in interactions and discussions with students, lack of adequate internet connectivity, students access to internet was difficult and expensive, and personal learning preference [37,38].There is need to cultivate a culture of change as institutions transition to online teaching, learning and assessment as this will reduce resistance. Advanced planning and infrastructure are important for successful implementation of e-learning [41]. There is also need to modulate the traditional methods of teaching, learning and assessment for e-learning.

Ninety three per cent (93.0%; n=27) reported that they believed in the benefits of online teaching of students
~~of~~

Formatted: Indent: Left: 0"

The majority of faculty did not agree that online teaching enhanced their teaching capacity while those who believed in the enhancement from online teaching felt that it added value to the learning of the students. They indicated the need for further training and adequate appropriate tools and reliable internet connectivity.

7 %(n=2) indicating that there were no benefits to students accruing from online teaching. ~~The majority faculty agreed that online teaching enhanced their teaching capacity while those who did not believe in the enhancement from online teaching felt that it added no value to the learning of the students. They indicated the need for further training and adequate appropriate tools and reliable internet connectivity.~~

The majority (83%; n=24) are already participating in online teaching with zoom being the most common platform used (62.1%) with social media and email being used to communicate with students. Other platforms were also used: Google meet, Google lessons, e-Mhare. In our faculty both synchronous (live or in real-time) and asynchronous (recorded or self-paced) are used through the University's learning management system (along with Zoom or Microsoft Teams). Synchronous e-learning is offered in the form of interactive teaching and clinical case discussions in small and large group formats [37] and asynchronous e-learning includes preparation of course materials for students in advances to be accessed later (recorded lectures, supportive videos, external links for recommended websites, and electronic books [37,41].

Literature indicates that there are many significant barriers to the adoption and implementation of e-learning by medical schools [5, 10]. These barriers include technology/infrastructure bar[illegible] student barriers [19]. In agreement with other studies from LMIC lack of infr[illegible] internet access, and poor quality of internet services (internet band width and [illegible] technical support, unfamiliarity with e-learning systems were mentioned as b[illegible] teaching/training and learning [15,19,20] : unavailability of university int[illegible] disrupted, broken down, interrupted power supply), absence of financial allow[illegible] data (high cost) , inattention by students, inability to perform skills transf[illegible] environment (working at home due to lockdown measures). The disturbances at [illegible] studies which showed that the learning environment at home posed the greate[illegible] literacy and competency was the least of their challenges [34].Low household i[illegible] for one to access the necessary equipment for online learning, along with poor power supply, poor internet connectivity, sociocultural dynamics as gender roles, educators' and learners' competence related to online teaching.

clara

clara
2022-05-04 03:40:25

In agreement. This has been rephrased as follows "The majority of faculty did not agreed that on-line teaching enhanced their teaching capacity while those who believed in the enhancement from on-line teaching felt that it added value to the learning of the students."

Blended teaching and learning, combining online and face-to-face teaching, balances benefits and drawbacks of online and face-to-face teaching [30]. Distance online course can prepare students as well as face-to-face. However, performance in online learning is reduced due to the COVID-19 driven social distancing and physical isolation as activities such as workshops, laboratories, and clinical rotations have had to be suspended [30].

The teaching environment poses challenges met by faculty inclusive of issues such as distractions at home (e.g., visitors, noise, and home chores) and limited space and facilities [34]. Poor internet connectivity, poor picture quality of videos and poor performing platforms were noted as possible barriers [25].Students and faculty need continuing training on online teaching and learning. A needs assessment should be performed among the students, health professions educators and the institution so that the identified needs and quality control be implemented at commencement of online teaching [35, 36, 42]. Poor internet connectivity, poor picture quality of videos and poor performing platforms were noted as barriers in this study as was noted in other studies [25].The needs assessment would help in the development of appropriate policies and a supportive culture.

Conclusions

The impact of the emergency embarking on online learning is not yet known since its e~~. E~~arly stages yet to assess the role of the academics on the future of e-learning in global health professions education. These findings can be used to improve health professions faculty approach to online teaching and learning as the global COVID-19 pandemic appears to be with us for a long time to come.

Institutions should improve the online teaching and learning infrastructures: learning management system (webinar facilities (zoom etc.) online learning platforms, mobile applications, Skype, Google Hangouts, training of both students and faculty on online teaching and learning, simulation/skills, laboratories, develop online curriculum, develop online examinations, telehealth, online video libraries. The developments should be embarked upon in line with their unique challenges of their faculty and the institutional existing online infrastructures. Continuing training and support on e-learning for faculty is important for successful acceptance and implementation of e-learning.

Methods of introducing and supporting health professions faculty transitioning to new teaching and learning circumstances and environments should provide contextualised both online and in-person strategies. Consideration of the negative and positive aspects of e-learning should allow for the development of a blended learning ~~approach ,~~approach, flipped class, team-based learning approach that integrates aspects of face-to-face learning and e-learning to achieve the intended learning outcomes especially in practical and clinical training.

Formatted: English (United Kingdom)

REFERENCES:

1. WHO (2020).WHO Director- General's Opening Remarks at the Media Briefing on COVID-19- 11 March 2020. Available online at: https://www.who.int/director-general/speeches/detail/who-director general-s-opening-remarks-at-the-media-briefinh-on-covid-19- 11-March-2020. (Accessed September 30:2021.

2. Watermeyer R, Crick T, Knight C, Goodall J. COVID-19 and digital disruption in UK universities: afflictions and affordances of emergency online migration. Higher Education.2021; 81:623-641.

3. The Lancet. Editorial 2020: Redefining vulnerability in the era of COVID-~~19.https://doi.org/10.1016/50140-~~19. https://doi.org/10.1016/50140- 6736 (20)30757-v

4. UNESCO COVID-19. Educational disruption and response 2020. (Available from https: //en. ~~unesco.~~unesco. o~~O~~rg /themes/education-emergenices/coronavirus-school-closure.

5. Rad FA, Otaki F, Baqain Z, et al. Rapid transition to distance learning due to COVID-19: Perceptions of postgraduate dental learners and instructors. PLOS ~~ONE.2021.https://doi.org/10.137/journal.pone.0246584~~ONE. 2021.https://doi.org/10.137/journal.pone.0246584.

6. Kernohan D. Which universities are moving to remote teaching? WONKHE 2020: https: // wonke.com/ blogs/which-universities-are-moving-to-remote-teaching/

7. Al Qhtani A, Al Swedan N, Almulhim A. et al. Online versus classroom teaching for medical students during COVID-19: measuring effectiveness and satisfaction. BMC Med Educ.2021; 21: 452-8.

8. Crawford J, Butler-Henderson K, Rudolph J, et al. M. COVID-19: 20 Countries' higher education intra-period digital pedagogy responses. ~~JAppl.Learn.Teach~~J Appl. Learn. Teach~~2020;3:1~~2020; 3:1 340-389.https;//journal.sfu.ca/jalt /index. php /jalt/index1-

9. Adman M, Anwar K. Online learning amid the COVID-19 pandemic: students 'perspectives. Journal of Pedagogical Sociology and Psychology.2020; 2: 45-51.

10. Kaur G. Digital life: Boon or bane in teaching sector on COVID-~~19.CLIO~~19. CLIO an Annual Interdisciplinary Journal of History.2020; 6: 416-427.

11. Schlenz MA, Schmidt A, Wostmann B et al.Students' and lecturers' perspectives on the implementation of online learning in dental education due to SARS-CoV-2 (COVID-19): a cross-sectional study. BMC Medical Educ2020; 20:354-361.

12. Ebner M, Schon S, Braun C et al. COVID-19 pandemic as E-learning boost? Chronological development and effects at an Austrian University against the background of the concept of "E-learning Readiness". Future Internet2020; 12:354-371.

13. Tabatabai S. Simulations and virtual learning supporting clinical education during the COVID-19 pandemic. Adv Medic Educ and Pract2020; 11:513-516.

14. Ahmed H, Alf M, Elghazaly ~~H.COVID~~H. COVID-19 and medical ~~education.Lancet~~education. Lancet.2020; 20:777-778.

15. Kiguli-Malwadde E. COVID-19 and its impact on health professions education in Africa. Afr Health.2020; 13:12-13.

16. Papapanou M, Routsi E, Tsamkis K et al. Medical education challenges and

innovations during COVID-19 pandemic. Postgrad Med J.doi:1136/postgrad 2021-140032.

17. Doherty J.Education and training update: e-learning in clinical teaching. Brit J Hosp Med. 2010; 71:44-47.

18. Wang Y, Rongbin Y, Liu Y et al.Qian W (2021). Students' and Teachers' perspective on the implementation of medical education in China: a qualitative study. Adv Med Educ Pract 12; 895-902.

19. Al-Balas M, Al-Balas H I, Jaber HM et.al. Distance learning in clinical medical education amid COVID-19 pandemic in Jordan: current situation, challenges, and perspectives 2020; 20: 341-8.

20. Gismalla MDA, Mohamed MS, Ibrahim OSO et. Al. Medical students' perception towards e- learning during

Formatted: Indent: First line: 0"

Formatted: Indent: First line: 0"

Formatted: Indent: First line: 0"

COVID-19 pandemic in a high burden developing country 2021; 21:3 77-13.

21. Olum R, Alulinda L, Kigozi E et.al. Medical education and e-learning during the COVID-19

21. pandemic: awareness, attitudes, preferences, and barriers among undergraduate medicine and nursing students at Makerere University, Uganda.2020; 7:1-9.

22. Li W, Gillies R, He M et al. Barriers and facilitators to online medical and nursing education during the COVID-19 pandemic: perspectives from international students from low- and middle- income countries and their teaching staff. Hum Resourc Health 2021;19:64-78.https://doi.org/10.1186/3129960-021-00609-9

23. Panahl P, Borna F.Distance learning challenges: challenges, new solution 2014 37th International Convention on Information and Communication Technology .Opatija: Electronics and Microelectronics (MIPRO); 2014. P653-6. https://doi.org/10.1109/MIPRO.20146859648.20.

24. Dawidziuk A, Kawkka M, Szyszka B et al. Global access to technology-enhanced medical education during the COVID-19 pandemic: the role of students in narrowing the gap. 2021; 9:S1-S5.

25. Di Giano P, Di Paolo C. COVID-19 and dental distance-based education: students' perception in an Italian University. BMC Med Educ.2021; 21:414-9.

26. Sigdel S, Ozaki A, Dhakal R, et al. Medical education in Nepal: Impact and challenges of the COVID-19 pandemic. Acad Med 2021; 96: 340-342.

27. Xin LJ, Hathim AAH, Yi NJ et al. Digital learning in medical education: comparing experiences of Malaysian and Japanese students. BMC Med Educ 2021; 21: 418-9.

28. Kiesewetter J, Sailer M, Jung VM et al. learning clinical reasoning how virtual patient case format and prior knowledge interact. BMC Medl Educ.2020;20;73.https://doi.org/10.1186/s12909-020-1987-y73. https://doi.org/10.1186/s12909-020-1987-y.

29. Dost S, Hossain A, Shehab M et al. Perceptions of medical students towards online teaching during the COVID-19 pandemic: a national cross-sectional survey of 2721 UK medical students. BMJ Open 2020;10:e042378.doi10: e042378.doi:10.1136/bmj/open-2020-042378.

30. Rossettini G, Geri T, Turolla A et al. Online teaching in physiotherapy education during COVID-19 pandemic in Italy: a retrospective case-control study on students' satisfaction and performance. BMC Med Educ 21;2021;21:4562021; 21:456-12.

31.Hugenholtz NI, de Groun EM,Smits PB et al. Effectiveness of e-learning in continuing Vallee A, BlacherJ,Carlou A. Sorbets E. Blended learning compared to traditional learning in medical education: systematic review w and d meta-analysis. J Med Internet Res.2020; 22(8)e16504.http: //doi.org/10.2196/16504. PMID:32773378; PMCID:PMC7445617.

32. Wilcha RJ. Effectiveness of virtual medical teaching during the COVID-19 crisis. Systematic Review.JMIR MedEdu.2020; 6(2):e20963.https://doi.org/10.21963.PMID:33106227:PMCID:PMC7682786): e20963.https://doi.org/10.21963.PMID:33106227:PMCID:PMC7682786.

33. Baherimoghadam T, Hamedani S, Mehrabi M et al. The effect of learning style and general self-efficacy on satisfaction of e-learning in dental students. BMC Med Educ 2021; 21: 463-9.

34. Barrot JS, Linares II, Del Rosario LS. Students' online learning challenges during the pandemic and how they cope with them: the case of the Phillippines. Educ Infor Tech. https://doi.org/10.1007/s10639-021-10589-x.

35. Ardekani A, Hosseini SA, Tabari P et al. Student support systems for undergraduate medical students during the COVID-19 pandemic: a systematic narrative review of the literature. BMC Med Edu. 2021; 21:352-64.

36. Ashoka B, Ong SY, Tay KH et al. Coordinated responses of academic medical centres to pandemics: sustaining medical education during the COVID-19 pandemic. Med Teacher. https://doi.or g/10.1080/01422159X. 2020. 1757634.

37. Zalat MM, Hamed MS, Bolbot SA. The experiences, challenges, and acceptance of e-learning as a tool for teaching during the COVID-19 pandemic among university medical staff. 2021. PLoS ONE 16(3):e0248758.https://doi.org/10.1371/journal.pone0248758): e 0248758. https://doi.org/10.1371/journal.pone0248758.

38. Hong KS, Lai KW, Holton D (2004). Students. Satisfaction and perceived learning with a web- based course.

Formatted: Indent: First line: 0"

Formatted: Indent: First line: 0"

J Educ Tech Soc 2004; 6:1-12.

39. Fischer SH, David D, Crotty BH et al. (2014). Acceptance and use of health information technology by community-dwelling elders. Int J Med Infor 2014; 83: 624-35.

40. Bolliger DU, Wasilik O. Factors influencing faculty satisfaction with online teaching and learning in higher education. Dist Educ 2009; 30: 103-16.

41. Almuwals A, Alqabbani S, Benajiba N et al. (2021). An emergency shift to e-learning in health professions education: a comparative study of perspectives between students and institution. Int J Learn Teach Educ Res2021; 20: 16-37.

42. Mishra L, Gupta T, Shree A .Online teaching-learning in higher education during lockdown period of COVID-19 pandemic 2020.Int Journal Edu Res Open. https://doi/10.1016/j.ljedro.2020.10012.

Formatted: Indent: First line: 0"

<u>Response to reviewers comments</u>

1. **How many were invited? Give the number please.**

   One hundred (100) faculty were invited. We used a convenient sample of 100 faculty from the above disciplines. The faculty approached where those who were accessible on campus during the time of data collection. Only 29 faculty responded.

2. **was the questionnaire validated? Totally how many questions were there related to the demographic information and how many related to online teaching and learning?**

   a. Yes. The questionnaire was developed by the researchers using relevant items from literature The tool was piloted on 5 faculty who were excluded from the main study. Following this pilot some items were removed and only relevant items were left: three 3 questions relating to demographic data and 13 questions relating to online teaching and learning were finally used for the study.

3. **This has already been described in the introduction.**

   Agreed, the passage has been removed.

4. **what is the relevance of this to the study carried out?**

   Not relevant to this section. This has been removed.

5. **What percentage of the total faculty members participated?**

   we got responses from 29 out of 100 members approached.

6. **It is saying the same thing as the previous paragraph**

   This will be removed

7. **As per the table 1, 48% agreed while 52% disagreed, that makes this presumption of "majority" of the faculty, invalid**

This has been rephrased accordingly: The majority of faculty did not agree that online teaching enhanced their teaching capacity while those who believed in the enhancement from online teaching felt that it added value to the learning of the students
