# Supplementary material for: Health professions faculty’s perceptions of online teaching and learning during the COVID-19 pandemic
Source: PLoS One. 2022 Nov 17;17(11):e0276170. doi: 10.1371/journal.pone.0276170 (PMC9671340; doi:10.1371/journal.pone.0276170)
Supplement: Attachment — Submitted filename: Response to Reviewers comments .docx [file pone.0276170.s003.docx]

**Response to reviewers comments**

1. **How many were invited? Give the number please.**

One hundred (100) faculty were invited. We used a convenient sample of 100 faculty from the above disciplines. The faculty approached where those who were accessible on campus during the time of data collection. Only 29 faculty responded.

2. **was the questionnaire validated? Totally how many questions were there related to the demographic information and how many related to online teaching and learning?**
   a. Yes. The questionnaire was developed by the researchers using relevant items from literature The tool was piloted on 5 faculty who were excluded from the main study. Following this pilot some items were removed and only relevant items were left: three 3 questions relating to demographic data and 13 questions relating to online teaching and learning were finally used for the study.
3. **This has already been described in the introduction.**

Agreed, the passage has been removed.

4. **what is the relevance of this to the study carried out?**

Not relevant to this section. This has been removed.

5. **What percentage of the total faculty members participated?**

we got responses from 29 out of 100 members approached.

6. **It is saying the same thing as the previous paragraph**

This will be removed

7. **As per the table 1, 48% agreed while 52% disagreed, that makes this presumption of "majority" of the faculty, invalid**

This has been rephrased accordingly: The majority of faculty did not agree that online teaching enhanced their teaching capacity while those who believed in the enhancement from online teaching felt that it added value to the learning of the students
