# Supplementary material for: Health professions faculty’s perceptions of online teaching and learning during the COVID-19 pandemic
Source: PLoS One. 2022 Nov 17;17(11):e0276170. doi: 10.1371/journal.pone.0276170 (PMC9671340; doi:10.1371/journal.pone.0276170)
Supplement: Attachment — Submitted filename: 9. Response to Reviewers comments .docx [file pone.0276170.s005.docx]

7. Response to reviewers’ comments

Please find below the response to the reviewers’ comments as requested in the emails sent on, **August 17, 2022** September **12, 2022 and September 20, 2022.**

1. Please note that funding information should not appear in the Acknowledgments section or Any other areas of your Manuscript. We will only publish funding information present in the Funding Statement section of the online submission form. Please remove any funding-related text from the manuscript.( **August 17, 2022**)

Response We have removed all the funding related texts from the manuscript

2. Your ethics statement should only appear in the Methods section of your manuscript. If your ethics statement is written in any section besides the Methods, please move it to the Methods section and delete it from any other section. Please ensure that your ethics statement is included in your manuscript, as the ethics statement entered into the online submission form will not be published alongside your manuscript. **(August 17, 2022**)

**Response The ethics statement has been revised in the manuscript (see manuscript with tract changes**

3. Please provide additional details regarding participant consent. In the Methods section, please ensure that you have specified (1) whether consent was informed and (2) what type you obtained (for instance, written or verbal). If your study included minors, state whether you obtained consent from parents or guardians. If the need for consent was waived by the ethics committee, please include this information. **(August 17, 2022**)

**Response The ethics statement has been revised in the manuscript indicating that a written consent was obtained on line and that no minors were involved in the study. (see manuscript with tract changes)**

1. Can you please upload an additional copy of your revised manuscript that does not contain any tracked changes or highlighting as your main article file.  This will be used in the production process if your manuscript is accepted.  Please amend the file type for the file showing your changes to Revised Manuscript w/tracked changes. **( September 12/2022)**

Response This was uploaded and is in the system: We uploaded two files Manuscript and Manuscript with tract changes as pointed out by the Editor

2. We note that several of your files are duplicated on your submission. Please remove any unnecessary or old files from your revision, and make sure that only those relevant to the current version of the manuscript are included **( September 12/2022)**

Response: This has been done as pointed out by the Editor. All the old documents were removed so that no duplication exists.

1. Please upload a Response to Reviewers letter which should include a point-by-point response to each of the points made by the Editor and / or Reviewers. (This should be uploaded as a 'Response to Reviewers' file type) **September 20/2022)**

Response. This has been uploaded as a Response to Reviewers as pointed out by the Editor

1. Upon checking you till haven't responded to the comments of the reviewers, please see the "Reviewer Comments to the Author" section in the Editor decision letter that was sent to you last June 14, 2022. (September 21, 2022)

**Response:** Please find below the response to the Editor decision letter that was sent to you last June 14, 2022.

1.How many were invited? Give the number please.

We used a convenient sample of 100 faculty from the above disciplines. The faculty

approached where those who were accessible on campus during the time of data

collection. Only 29 faculty responded.

2.was the questionnaire validated? Totally how many questions were there related to

the demographic information and how many related to online teaching and learning?

The tool used to collect data was piloted on 5 faculty who were excluded from the main

study. Following this pilot some items were removed and only relevant items were left:

three 3 questions relating to demographic data and 13 questions relating to online

teaching and learning were finally used for the study.

Powered by Editorial Manager® and ProduXion Manager® from Aries Systems Corporation

3.This has already been described in the introduction.

This will be removed.

4.what is the relevance of this to the study carried out?

This will be removed.

5.What percentage of the total faculty members participated?

we got responses from 29 out of 100 members approached.

6.It is saying the same thing as the previous paragraph

This will be removed

7.As per the table 1, 48% agreed while 52% disagreed, that makes this presumption of

"majority" of the faculty, invalid

This has been rephrased accordingly: The majority of faculty did not agree that online

teaching enhanced their teaching capacity while those who believed in the

enhancement from online teaching felt that it added value to the learning of the

students
